# Effects of Exercise on the Resting Heart Rate: A Systematic Review and Meta-Analysis of Interventional Studies

**DOI:** 10.3390/jcm7120503

**Published:** 2018-12-01

**Authors:** Anne Kerstin Reimers, Guido Knapp, Carl-Detlev Reimers

**Affiliations:** 1 Faculty of Behavioral and Social Sciences, Institute of Human Movement Science and Health, Technical University of Chemnitz, Straße der Nationen 62, D-09111 Chemnitz, Germany; 2 Department of Statistics, TU Dortmund University, Vogelpothsweg 87, D-44227 Dortmund, Germany; guido.knapp@tu-dortmund.de; 3 Paracelsus-Klinik Bremen, In der Vahr 65, D-28329 Bremen, Germany; c.d.reimers@outlook.de

**Keywords:** sports, physical activity, trail, cardiovascular health, PRISMA

## Abstract

Resting heart rate (RHR) is positively related with mortality. Regular exercise causes a reduction in RHR. The aim of the systematic review was to assess whether regular exercise or sports have an impact on the RHR in healthy subjects by taking different types of sports into account. A systematic literature research was conducted in six databases for the identification of controlled trials dealing with the effects of exercise or sports on the RHR in healthy subjects was performed. The studies were summarized by meta-analyses. The literature search analyzed 191 studies presenting 215 samples fitting the eligibility criteria. 121 trials examined the effects of endurance training, 43 strength training, 15 combined endurance and strength training, 5 additional school sport programs. 21 yoga, 5 tai chi, 3 qigong, and 2 unspecified types of sports. All types of sports decreased the RHR. However, only endurance training and yoga significantly decreased the RHR in both sexes. The exercise-induced decreases of RHR were positively related with the pre-interventional RHR and negatively with the average age of the participants. From this, we can conclude that exercise—especially endurance training and yoga—decreases RHR. This effect may contribute to a reduction in all-cause mortality due to regular exercise or sports.

## 1. Introduction

A major goal in healthcare is to increase life expectancy and eminently improve healthy and happy aging by compressing morbidity into a shorter period in a later stage of the lifespan [1]. Life expectancy has increased rapidly during the last century and disability-adjusted life expectancy has been extending as well. Regular exercise and physical activity throughout a lifespan can improve life expectancy [2,3] and disability-adjusted life expectancy, as shown in many studies [4,5].

One possible mechanism explaining increases in life expectancy through exercise and physical activity might be the mediating effect of resting heart rate (RHR): possibly, regular exercise and/or physical activity cause a reduction in RHR [6,7,8], and RHR seems to be inversely related with life expectancy [9] and positively related with cardiovascular and all-cause mortality [10]. The relationship between RHR and life expectancy has been studied in mammals by Levine [11], who found a semi-logarithmic relationship in mammals and concluded that the total number of heartbeats per lifetime is remarkably constant. Furthermore, he calculated that the inverse relationship of RHR and life expectancy in mammalians could be expressed mathematically with on average 7.3 ± 5.6 × 10^8^ heart beats per lifetime. Only humans fall out of the alignment and have reached a life expectancy of about 80 years [11]. This might be due to advances in science, medicine, and sociology. However, in humans, RHR also seems to be an indicator of mortality, which has been analyzed in some studies [9,12,13].

Thus, the examination of effects regular exercise and physical activity on RHR is of particular interest, and has been studied in previous systematic reviews: Cramer, Lauche, Haller, Steckhan, Michalsen, and Dobos [8] found a significant reduction in heart rate through yoga interventions in their systematic review of randomized controlled trials on effects of yoga on diverse cardiovascular risk factors. Their meta-analysis revealed a reduction in heart rate of 6.59 bpm in studies that compared yoga with no-treatment usual care, or any active treatment in healthy participants. In another meta-analysis encompassing 13 studies, Huang, Shi, Davis-Brezette, and Osness [6] concluded that endurance training causes RHR reductions of 8.4% in older individuals which were even higher in controlled clinical trials with a training length of more than 30 weeks. Furthermore, a decrease in heart rate at quiet condition was found after tai chi exercise in healthy adults as shown in the meta-analysis of Zheng, Li, Huang, Liu, Tao and Chen [7]. However, to the best of our knowledge, a comprehensive review and meta-analysis of the effects of regular physical exercise on the RHR in various sports and exercise and a comparison of the effects of different exercise and sports is still missing.

Thus, the aim of this systematic literature review was to calculate the effects of any regular sports and/or exercise on RHR of healthy subjects through meta-analysis by considering different types of sports and exercise and by considering differences between men and women.

## 2. Materials and Methods

The review was performed adopting the recommendations of the Preferred Reporting Items for Systematic Reviews and Meta-Analyses (PRISMA) guidelines [14].

### 2.1. Search Strategy

Six electronic databases were searched on 13 May 2018: Google Scholar (the first 500 citations ordered by relevance), LIVIVO (the search engine of the German Central Library of Medicine), PubMed, Scopus, Springer Link, and Web of Science. If available, the search term has been applied in the search field “all fields”, and the “in humans” filter has been used (if available). The search term applied was: “(resting heart rate or resting pulse rate or resting heart frequency or resting pulse frequency) and (sport or training or exercise or yoga or tai chi or qigong) and control*”.

### 2.2. Study Selection

Only three relevant journal articles presented titles including the search term “resting heart rate” (or synonyms of this term). Moreover, in many of the relevant articles, RHR was presented as a less important outcome instead of the primary outcome and has not been mentioned in the abstract or even as a keyword. Nevertheless, to ensure identification of relevant primary studies through the study selection process, both the title and the abstract—and additionally, if available, the keywords—were considered in the first step of the screening process. All relevant references that could not be conclusively excluded were kept for further screening.

Next, the abstracts of all articles (if available) were checked regarding the characteristics of the participants, study design, and the intervention program. If the abstract indicated that the study potentially fulfilled the eligibility criteria or did not provide sufficient information for decision-making, the full text of the article was screened in a third step. Afterwards, articles presenting (nearly) the same content regarding the participants and results have been judged as double publications.

**Figure 1:**
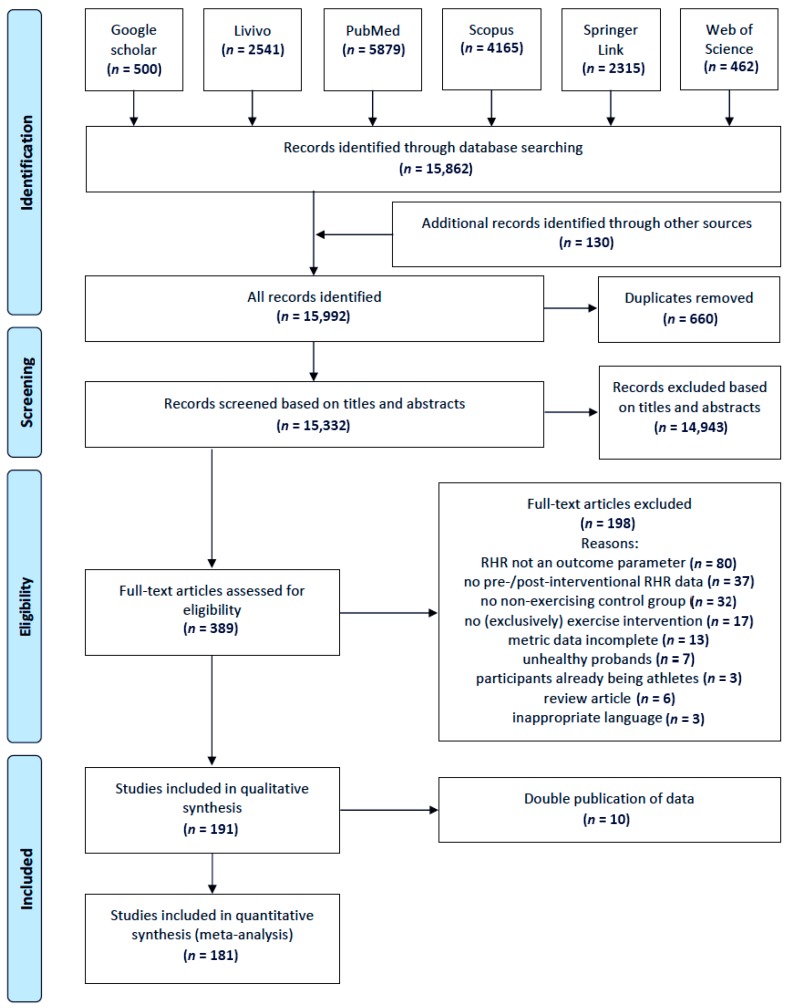
Flow chart of the screening and selection process for the identification of studies for inclusion in the present systematic review according to PRISMA (Moher et al. [14]).

Finally, the references cited in the former systematic reviews and meta-analysis on the topic at hand [6,7,8,15] as well as cited by two own meta-analyses focussing on the effects of sports interventions on the arterial blood pressure and/or lipids [16,17] were screened and considered for inclusion (additional records identified through these sources: *n* = 130; cf. Figure 1). The screening process has been conducted by one reviewer. If necessary, a second reviewer was consulted for deciding on eligibility of the reference.

### 2.3. Eligibility Criteria

The following eligibility criteria have been defined a priori to the study selection process. Only primary studies that fulfilled all of the eligibility criteria were included in the present systematic review.

#### 2.3.1. Types of Studies

Only controlled trials (randomized or non-randomized) were considered, and the study had to be published as a peer-reviewed journal article written in English, German, French, Spanish, or Portuguese language. Additionally, if an English language abstract and RHR data presented in English in the full text article were available, the translation of the article was considered. Thus, one Japanese article [18] has been translated and could be included in the present review.

#### 2.3.2. Types of Participants

Studies with individuals being pregnant or having diseases like diabetes mellitus, metabolic syndrome, or a limited physical mobility due to diseases (e.g., cardiac and musculoskeletal disorders) were excluded whereas studies with participants that were overweight, obese, or had arterial hypertension or dyslipidaemia were included. If the individuals in the study were regularly exercising at the beginning of the study, the study was not excluded due to this fact.

#### 2.3.3. Types of Interventions

The intervention should have comprised any type of sports and/or exercise including aerobic and anaerobic (e.g., high intensity interval training), endurance (including ball and team sports) or strength training, school sports, yoga, qigong, or tai chi.

#### 2.3.4. Types of Outcome Measures

For inclusion in the meta-analyses, the trials had to report the RHR before and after the intervention (pre/post-measurements), presenting the mean values plus scale parameters (standard deviation or standard error or the 95% confidence interval).

### 2.4. Data Management and Data Extraction

After checking the potentially relevant primary studies according to the eligibility criteria mentioned above, the articles were additionally checked for double publication. The following data was extracted from each primary study included in this systematic review: author(s); year of publication; number, gender, and age of the participants; type of sports/exercise; duration of the intervention; frequency of exercise sessions; method of RHR measurement; randomisation; blinding; RHR at the beginning and end of the interventional period in the intervention and control groups, resp.

In case of studies with more than two arms (one control group and more than one exercise group), those comparisons with the higher exercise intensity, duration or frequency, continuous (versus interval) and concentric (versus eccentric) strength training, land-based (versus aquatic) training, and jogging/running (versus soccer) were considered for the meta-analyses. Additionally, if both pre and post-menstrual or pre and post-menopausal RHR results were presented, the first ones were included in the meta-analyses.

### 2.5. Risk of Bias Assessment

The methodological quality of the studies included was evaluated by one reviewer by considering three aspects: (1) the mode of randomization of the study participants to the intervention and control groups, (2) possible blinding of the examiners, and (3) the mode of assessing RHR have been registered (Table 1).

### 2.6. Statistical Analyses

#### 2.6.1. Statistical Methods for the Meta-Analyses

The meta-analysis was carried out using the meta and metafor packages in the freely-available statistical R software, version 3.4.4 [19]. Most studies reported mean and standard deviation or standard error of RHR before and after the intervention separately for intervention and control group. When confidence intervals for the mean or the mean difference were reported, we calculated the relevant standard errors. We chose the standardized mean difference (SMD) as the primary effect size. For both groups we firstly calculated the difference between the mean RHR after and before the intervention, and then calculated the difference of these differences between intervention and control group divided by an appropriate standard deviation—either the common standard deviation of the two groups or a suitable combination of both individual standard deviations. Consequently, negative values are in favor of the intervention group for reducing the mean RHR. The random-effects meta-analysis was used for combining the study-specific results. We report the classical meta-analysis confidence interval as well as the modified confidence interval proposed by Hartung and Knapp [20]. Two-sided *p*-values < 0.05 are considered as significant. We judge the heterogeneity with the homogeneity test statistics denoted by Cochran’s *Q* and Higgins–Thompson *I*^2^, a measure which judges the impact of heterogeneity.

Additionally, we calculated the meta-analytical mean values of the RHR before and after intervention, separately for both groups using random-effects meta-analysis approach. Finally, relative changes defined as (mean after intervention minus mean before intervention)/(mean before intervention) were combined in the random-effects meta-analysis model for both groups.

#### 2.6.2. Additional Statistical Methods

The Bravais–Pearson correlation coefficients r between the age of the participants, number, and frequency of the sportive interventions, and the pre-interventional RHR of the training group have been calculated on the one hand and the extent of the change of the RHR due to physical activity on the other. Two-sided p-values of <0.05 were considered as significant.

## 3. Results

### 3.1. Studies, Participants, and Exercise

Initially based on database queries and through screening of additional sources 15,992 articles have been identified and included in the screening process. After exclusion of 660 duplicates, 15,332 articles have been screened. Finally, the literature search yielded 191 studies meeting the eligibility criteria. Ten of these articles presented the same data as presented in another article and therefore were excluded. Thus, finally the data of 181 articles encompassing 215 samples were included in the meta-analytical synthesis. The selection process of the articles included in this systematic review is presented in Figure 1 and detailed descriptions of the 215 samples included in the meta-analysis are presented in Table 1.

All studies included have been published between 1971 and 2018. Altogether, 12,952 individuals were incorporated in the intervention (*n*_I_ = 6763) and control groups (*n*_C_ = 6189). The sample sizes of these groups ranged from 5 to 1456 within the studies with a median sample size of 17 participants (interquartile range, 12 to 26.5 participants). Of the 215 comparisons 92 included both female and male participants, whereas 65 only included females and 58 only males. Wiley et al. [21] did not report the gender of their participants (denoted as ‘human subjects’ only). This study was considered in the group of studies including both sexes.

Of the 191 studies presenting 215 samples, 121 looked into the effects of endurance training, 43 strength training, 15 combined endurance and strength training, and 5 additional school sports. The interventions included the following sports types: 21 yoga, 5 tai chi, 3 qigong, and 2 unspecified sport types. The exercise interventions lasted between 2 weeks and 2 years (median value, 12 weeks, interquartile range, 8 to 16.75 weeks). The participants exercised 0.8 to 7 times per week (median value, 3 times, interquartile range, 3 to 3.6 times per week). Altogether, they completed between 7 and 312 exercise sessions (median value, 36 sessions, interquartile range, 24 to 54 sessions) during their participation in the intervention studies.

### 3.2. Effects of Exercise on RHR by Considering Different Types of Sports/Exercise

The mean baseline and post-interventional RHR according to the different forms of sports and/or exercise are presented in Table 2. Under consideration of all comparisons, the RHR significantly decreased more in the exercising groups (intervention groups) compared to the control groups (all studies: −4.7% and −3.3 bpm resp., females only: −4.8% and −3.4 bpm, resp., males only: −6.4% and −4.3 bpm, resp., studies including both females and males: −3.6% and −2.6 bpm, resp.). The meta-analyses on specific types of sports and exercise also revealed significant higher decreases in RHR in the intervention compared to the corresponding control groups for endurance training (all groups), yoga (females and males only), strength training (females only), and combined endurance and strength training (females only, not Hartung-Knapp CI).

### 3.3. Additional Analyses

The extents of decrease of the RHR due to the interventions were significantly associated with the initial RHR (all studies: *r* = 0.30, *n* = 215, *p* < 0.001; women: *r* = 0.28, *n* = 65, *p* = 0.024; men: *r* = 0.22, *n* = 59, *p* = 0.09; both sexes: *r* = 0.39, *n* = 91, *p* < 0.001). The average age of the study participants was not related with the baseline RHR of the participants (*r* = −0.14, *n* = 194, *p* = 0.052; 21 trials did not mention the mean age of the participants), but with the sport/exercise-induced decrease of the RHR (*r* = 0.15, *n* = 194, *p* = 0.037). The higher the average age the smaller was the decrease of the RHR. The average age of the exercising participants was not significantly associated with the number of training sessions throughout the intervention (*r* = 0.133, *n* = 194, *p* = 0.064). The number of exercise sessions throughout the interventions did not significantly influence the changes in the RHR (*r* = 0.088, *n* = 215, *p* = 0.20).

### 3.4. Risk of Bias in Individual Studies

The characteristics of the samples and the results of the risk of bias assessment included in the present review are shown in Table 1. In 178 (82.8%) samples, the participants were randomly assigned to the exercising and control groups with 10 articles presenting detailed information about the mode of randomization. Nine (4.2%) samples were based on non-randomized collectives. In the remaining 27 (12.6%) samples, no information about randomization was given.

In only two studies [86,161], the researchers were blinded regarding the assignment of the participants to the intervention and control group.

In 153 (71.2%) samples, the method used for measuring the RHR was described: conventional or long-term electrocardiography or sport tester heart rate monitors (*n* = 111), automatic sphygmomanometer (*n* = 25), auscultation (*n* = 8), pulse monitor (*n* = 4), palpation (*n* = 3), oscillometer (*n* = 1), and oximeter (*n* = 1). 62 studies gave no information regarding the measurement methods of the RHR.

## 4. Discussion

### 4.1. General Discussion of Findings

The present meta-analysis is the first determining the effects of any regular physical activity, exercise, or sports on RHR of healthy people by considering different types of sports and exercise as well as differences between males and females. The literature search in six data bases and additional sources revealed a total of 191 primary studies suitable for inclusion that overall encompassed 215 samples, resulting in a comprehensive evaluation of existing studies on the effects of sports and exercise on RHR in males and females.

### 4.2. Effects of Different Types of Sports on RHR

In the meta-analysis of 16 trials by Huang et al. [6], the magnitude of net change of RHR due to endurance training in older adults averaged 6.16 ± 0.97 bpm, representing a mean reduction of 8.4%. The mean effect of yoga identified in the meta-analysis of nine trials by Cramer et al. [8] was very similar (M: −6.59 bpm, 95%-CI: −12.89 to −0.28 bmp). This meta-analysis included both healthy and diseased participants. Zou et al. [15] combined four trials and described a significant decrease of the RHR due to qigong (SMD: −0.87, no absolute difference of RHR presented). Tai chi exercise reduced the RHR by 0.72 (SMD: Cl = −1.27 to −0.18, *n* = 6 trials) [7].

Our meta-analyses indicate that endurance training as well as yoga decrease the RHR. After endurance training, the mean decrease of RHR of the exercising participants as compared to the non-exercising was depending on the sexes of the participants (4.5% to 9.0% and 2.7 to 5.8 bpm, resp.). These results are a little bit smaller than those found by Huang et al. [6] who meta-analytically combined 13 studies with a total of 410 exercising individuals. Their overall net change in RHR amounted to −6.16 ± 0.97 bpm (M ± SE, 95%-CI: −8.15 to −4.18, *p* < 0.001). Our results on effects occurring after a yoga program indicate a mean decrease of the RHR due to the exercise (4.1% to 5.5% and −5.2 to −5.5 bpm, resp.). However, the reduction found in our study was slightly smaller than that found by Cramer et al. [8] in controlled clinical trials (M = −6.59 bpm, 95%-CI: −12.89 to −0.28 bpm). The results of Zheng et al. [7], indicating a significant decrease of RHR due to tai chi could not be confirmed in the present meta-analysis. However, in contrast to our approach, the authors included only two randomized controlled trials, eight non-randomized controlled trials, three self-controlled trials, and seven cohort studies. Thus, the basis of their meta-analysis differed considerably with the present one.

The meta-analysis of the five RCTs on strength training included in our review did not yield significant effects: our results indicate that strength training has no significant impact on the RHR. Nevertheless, by stratification of sexes strength training (−5.0% and −2.2 bpm) and a combined endurance and strength training (−4.5% and −3.8 bpm) significantly decreased the RHR in females, but not in males.

All other sports types also resulted in a decrease of the RHR. However, the effects did not reach significance, due possibly to insufficient statistical power caused by too small sample sizes and/or only few available trials. The higher the initial RHR, the more the RHR decreased due to exercise. The effect occurs after only a few months—on average, three months with three training sessions per week. Furthermore, the participants´ age was negatively associated with the exercise-induced decrease of the RHR, although the elderly participants did not exercise less than their younger counterparts.

### 4.3. Possible Mechanisms of the Heart Rate-Decreasing Effect of Sports and Physical Activity

Bahrainy et al. [211] suggest that neither an increase in resting parasympathetic tone nor a decrease in response to beta-adrenergic stimulation contribute to the decrease in RHR after regular exercise or physical activity in humans. The effect may be due to a decrease in the intrinsic heart rate via mechanisms which have not yet been fully understood. In the case of yoga, lower RHR may also be caused by an enhanced parasympathetic output [212].

### 4.4. Possible Relevance of Exercise-Induced Reductions of the RHR for Mortality

Recently, Aune et al. [10] calculated that an increase of the RHR by 10 bpm increases all-cause mortality by 17%. The evidence regarding negative influence of elevated RHR on cardiovascular and thus all-cause death is constantly increasing. The mechanisms of this relationship are still not completely known. Possible mechanisms may be endothelial dysfunction, reduced artery compliance and distensibility, and consequently increased arterial wall stress and elevated pulsed wave velocity, which is further associated with increased after load and ultimately systemic hypertension [213]. However, the RHR does not fulfil all criteria for being an independent risk factor, as scientific evidence proving that treating an elevated RHR reduces mortality is still lacking [214]. Thus, it cannot be excluded that the relationship between the RHR and mortality may be non-causative. If there is a causative relationship assuming a persistently reduced RHR due to lifelong endurance—according to the results of our meta-analysis—exercise and yoga could reduce mortality by about 4.4% to 8.9% and about 6.0% to 8.7%, respectively, depending on the sex of the athlete.

### 4.5. Risk of Bias

The majority of studies randomly assigned the participants to the intervention and controls groups. It is possible that the percentage may be even considerably higher, since only nine studies have been declared as non-randomized, whereas the remaining 27 studies did not report on randomization. However, including non-randomized interventional studies in the analysis probably does not affect the results, since the RHR as an autonomic function is unlikely to be influenced by the participants wills.

In only two studies, the researcher was blinded regarding the assignment of the participants to the intervention or control group. Double blinded exercise and sport studies are difficult to perform. However, this could substantially influence the measurements of the RHR. Additionally, the accuracy of the RHR measurements might be lower for physical assessments (like palpation or auscultation) than for electrocardiography. Thus, the accuracy of measuring the RHR differs between the studies that used different methods. However, a systematic error due to different measurement methods applied in the included studies is rather unlikely, given that within each study the method did not change. Furthermore, the RHR is not a stable construct and may vary within short time periods. Consequently, the point in time the measurements were conducted could have influenced the identified values. Nevertheless, it is hardly imaginable that any systematic errors occurred which influenced only the training or only the control group.

In the excluded studies, outcome reporting bias and publication bias may exist: for example, several studies examining the relationship between physical activity and/or sport and health-related outcomes probably involved and measured RHR but did not report the results. However, as in the studies included in the present meta-analysis, only in one study was RHR the primary outcome [204]—hence we assume that non-reporting of RHR does not distort our results for RHR.

Only studies presenting complete outcome parameters (RHR before and after the intervention as well as scattering data) were included in the meta-analysis. Several studies do not present the mean or median age of their participants as well as the corresponding scattering data. Lacking detailed information on the participants´ ages and gender as in the study of Wiley et al. [21] also do not affect the effect of training on the RHR.

### 4.6. Strength and Limitations

A strength of this systematic review and meta-analysis is the systematic search of primary studies employing six search engines and encompasses more than 200 samples. Additionally, types of sports and exercise performed in the intervention were considered to enable the identification of sport-specific effects on RHR. However, this meta-analysis also consists of several limitations. First, the duration and frequency of the sports and exercise conducted during the intervention programs varied considerably—even when summarizing interventions of the same types of sports. Second, the effects of the different types of sport and exercise (endurance or strength training, Asian sports activities) cannot be compared to each other due to different physiological effects. Thus, the results of our analysis only showed if the different types of sports and exercise had an impact on the RHR or not. Third, the number of studies examining school sports, tai chi, and qigong was too low to draw final conclusions, resulting in the impossibility of calculating the effects associated with these activities.

## 5. Conclusions

Our meta-analyses indicate that both endurance sports as well as yoga decrease the RHR. These types of sports activities—after exclusion of otherwise treatable disease such as anemia, cardiac or endocrine disorders (e.g., hyperthyroidism)—could be used as a treatment in case of unfavorable high RHRs. The positive correlation between the RHR-decreasing effect and the initial RHR indicates that this therapeutic approach could be especially interesting in case of tachycardia. However, further studies are needed focusing on effects of Tai Chi and Qigong which have only been considered in a few studies. Furthermore, future studies could use shorter time intervals between measurements of RHR to get insights into the underlying processes, contributing to the reductions in RHR.

In summary, exercise-related decrease of RHR may contribute to—or at least indicate—increasing life expectancy. However, this has not been investigated in the current meta-analysis and should therefore be a topic of further studies.

## Figures and Tables

**Table 1:** Descriptive characteristics of the samples included in the meta-analyses.

Author(s)	Sex	N (IG)	Age * (yr)	Exercise in the IG			Measurement of RHR	Randomization	Blinding	IG		CG		Rel. RHR Change IG/CG (%)
				Duration (/wk)	FreQuency (/wk)	Overall Number of Units				Baseline RHR (/min)	Final RHR (/min)	Baseline RHR (/min)	Final RHR (/min)	
**Endurance training**														
Abadi et al. [22]	♀ + ♂	25	22.6	12	3	36	ECG	random	n.p.	73.96	72.44	73.88	73.72	−1.8
Akinpelu [23]	♂	10	55.7	10	4	40	n.p.	n.p.	n.p.	73	72.8	81.8	78.6	3.8
Akwa et al. [24]	♀	8	61.25	8	3	24	ASP	random	n.p.	76.5	72	83	78.5	−0.5
Andersen et al. [25]	♂	13	46.7	17	2	34	ASP	random	n.p.	79	67	72	73	−16.4
Anek & Bunyaratavej [26]	♀	26	50.67	4	3	12	n.p.	random	n.p.	79.26	75.85	79.33	79.53	−4.5
Anek et al. [27]	♀	15	39.86	16	3	48	ASP	random	n.p.	78.26	76.8	79.13	79.56	−2.4
Apekey et al. [28]	♀ + ♂	12	18–38	8	2	16	ECG	random	n.p.	85	78	83	76	0.2
Aweto et al. [29]	♀ + ♂	23	44.1	4	2	8	auscultation	random	n.p.	77.2	70.9	75.4	73.1	−5.3
Baker et al. [30,31]	♀ + ♂	39	47.3	12	3	36	ASP	IVRS	n.p.	68.6	69.5	67.9	68.9	−0.2
Beck et al. [32,33]	♀ + ♂	9	20.1	8	3	24	n.p.	random	n.p.	64	56	60	58	−9.5
Blum et al. [34]	♀	21	32.8	12	4	48	n.p.	not random	n.p.	80.1	71.4	69.3	72.4	−14.7
Blumenthal et al. [35,36]	♀ + ♂	33	67	17	3	51	ECG	random	n.p.	74	72	70	69	−1.3
Bocalini et al. [37]	♀	25	64	12	5	60	ECG	random	n.p.	90	85	90	89	−4.5
Braith et al. [38]	♀ + ♂	14	65	26	3	78	n.p.	random	n.p.	69	63	65	66	−10.1
Boutcher & Stein [39]	♂	25	46.2	8	3	24	ECG	random	n.p.	71.3	67.9	73.1	77.3	−10.0
Broman et al. [40]	♀	15	69	8	2	16	ECG	random	n.p.	78.7	72.2	69.4	71.1	−10.5
Carroll et al. [41]	♀ + ♂	29	68.4	26	3	78	ECG	random	n.p.	63.4	61.8	61	60.3	−1.4
Ciolac et al. [42]	♀	11	24.4	16	3	48	n.p.	random	n.p.	80	77	79	79	−3.7
Connolly et al. [43]	♀	13	39	16	2	32	ASP	random	n.p.	77	73	77	74	−1.4
Connolly et al. [44]	♀	15	44	12	3	36	n.p.	random	n.p.	70	65	68	69	−8.5
Cononie et al. [45]	♀ + ♂	11	72	8	3	24	ECG	n.p.	n.p.	66	61	68	67	−6.2
Cononie et al. [45]	♀ + ♂	6	72	8	3	24	ECG	n.p.	n.p.	66	62	66	65	−4.6
Davies & Daggett [46]	♀	8	29.2	8	3	24	n.p.	n.p.	n.p.	64.9	64.8	79.3	76.4	3.6
Delecluse et al. [47]	♂	20	64.5	20	2.5	50	ECG	random	n.p.	78.2	68.9	73.3	73.9	−12.6
Dowdy et al. [48]	♀	18	31.5	10	3	30	n.p.	non random	n.p.	67.1	61.7	71.8	73.3	−9.9
Duey et al. [49]	♀	16	23.6	6	3	18	ECG	n.p.	n.p.	80.7	77.1	78	78	−4.5
Duncan et al. [50]	♂	44	30.4	16	3	48	ECG	random	n.p.	73.7	65.2	74.3	73.8	−10.9
Ewart et al. [51]	♀	44	Adol.	18	n.p.	n. c.	ECG	random	n.p.	79.7	79.2	83.8	83.8	−0.6
Finucane et al. [52]	♀ + ♂	48	71.4	12	3	36	ECG	random	n.p.	66	62.1	65.2	64.4	−4.7
Geenen et al. [53]	♀ + ♂	38	6.8	8	4	32	ECG	random	n.p.	84	89	85	84	7.2
Goldberg et al. [54]	♂	15	20.5	4	3	12	ECG	random	n.p.	69.4	61	63.6	59.2	−5.6
Gormley et al. [55]	♀ + ♂	13	21	6	3	18	ECG	random	n.p.	64	65	67	67	1.6
Gossard et al. [56]	♂	23	49	12	5	60	ECG	random	n.p.	68	66	74	74	−2.9
Gutin et al. [57]	♀ + ♂	17	9.6	17	5	85	ECG	random	n.p.	81	77.5	85	86.8	−6.3
Hagberg et al. [58]	♀ + ♂	11	64	37	2.5	92.5	n.p.	random	n.p.	73	65	75	76	−12.1
Halbert et al. [59]	♀ + ♂	149	67.3	52	3	156	n.p.	random	n.p.	70.9	71.1	71.3	71.6	−0.1
Hamdorf & Penhall [60]	♀	18	82.4	26	2	52	ECG	random	n.p.	74.4	71.8	72.7	75.4	−7.0
Hamdorf et al. [61]	♀	27	64.8	26	2	52	n.p.	random	n.p.	76	74	76	74	0.0
O’Hartaigh et al. [62]	♀ + ♂	213	76.8	52	3	106	n.p.	random	n.p.	69	66	68	66	−1.4
Hespel et al. [63]	♂	13	39.2	16	3	48	ECG	random	n.p.	65	58.2	66	64.8	−8.8
Hewitt et al. [64]	♀ + ♂	12	41	8	4	32	ECG	random	n.p.	66	63.8	67	65.3	−0.8
Heydari et al. [65,66]	♂	25	24.7	12	3	36	ECG	random	n.p.	62.2	57.9	62.7	63.7	−8.4
Heydari et al. [67]	♂	17	24.4	12	3	36	ECG	random	n.p.	67	61	69	70	−10.3
Higashi et al. [68]	♀ + ♂	20	53	12	6	72	n.p.	random	n.p.	71.8	68.8	73.1	69.2	1.2
Hill et al. [69]	♀ + ♂	87	64	10.5	4	42	ECG	n.p.	n.p.	85.03	76.94	79.15	78.89	−9.2
Hiruntrakul et al. [70,71]	♂	19	21	12	1	12	n.p.	random	n.p.	88	78	90	93	−14.2
Hornstrup et al. [72]	♀	14	23.9	12	1.7	20.4	ASP	random	n.p.	59	57	61	62	−4.9
Jahromi et al. [73]	♀	30	10–11	8	3	24	pulse monitor	n.p.	n.p.	81.12	72.31	80.05	80.26	−11.1
Jurca et al. [74]	♀	49	PMP	8	3.5	28	ECG	random	n.p.	68.1	65	66	65.8	−4.3
Karavirta et al. [75]	♂	23	55.6	7	1	7	ECG	random	n.p.	61	57	54	53	−4.8
Karavirta et al. [76]	♀	26	52	7	1	7	ECG	random	n.p.	63	60	65	62	−0.2
Kiens et al. [77]	♂	24	40	12	3	36	ECG	random	n.p.	69.4	59.3	64.9	60.7	−8.6
Kim et al. [78]	♂	14	17	6	5	30	n.p.	random	n.p.	75.1	70.4	76.3	76.2	−6.1
Knoepfli-Lenzin et al. [79]	♂	15	36	12	2.5	30	n.p.	random	n.p.	70	61	74	68	−5.2
Kokkinos et al. [80]	♂	23	57	16	3	48	n.p.	random	n.p.	73	71	73	75	−5.3
Korshoj et al. [81,82]	♀ + ♂	57	44.9	52	2	104	ECG	c.r.	n.p.	71.7	69.98	70.5	70.3	−2.1
Krustrup et al. [83]	♂	12	20–43	12	2.5	30	ECG	random	n.p.	59	53	60	61	−11.6
Krustrup et al. [84]	♀	21	37	16	1.8	28	ECG	random	n.p.	62	57	63	63	−8.1
Krustrup et al. [85]	♀ + ♂	46	9.4	10	2.1	21	ECG	random	n.p.	70.4	69.9	70.6	71.2	−1.5
Lamina [86]	♂	112	58.63	8	3	24	ASP	random	double blind	82.25	70.78	82.6	80.5	−11.7
Linder et al. [87]	♂	21	11–17	8	4	32	n.p.	random	n.p.	72	74.8	69.7	75.7	−4.3
Loimaala et al. [88]	♂	26	46.8	22	5	110	ECG	random	n.p.	68	64	67	69	−8.6
Mahdiabadi [89]	♂	10	18.5	8	3	24	auscultation	random	n.p.	68.2	66.2	71.3	68.3	1.3
Melanson & Freedson [90]	♂	11	36.6	16	3	48	ECG	non random	n.p.	64.9	59.8	61	60.5	−7.1
Menezes-Cabral et al. [91]	♀	53	64.8	17	4	68	ECG	n.p.	n.p.	75.2	71.6	77.6	78.7	−6.1
Menezes-Cabral et al. [91]	♂	51	64.5	17	4	68	ECG	n.p.	n.p.	77.1	72.8	87	87.9	−6.5
Meucci et al. [92]	♀ + ♂	6	9.9	4	5	20	ECG	random	n.p.	97	80	104	99	−13.4
Miyai et al. [93]	♂	17	46.6	12	3	36	ECG	random	n.p.	68.5	57	66.7	63	−1.9
Mogharnesi et al. [94]	♂	12	14.32	8	3	24	n.p.	random	n.p.	76.4	69.75	74.6	75.6	−9.9
Mohr et al. [95]	♀	21	44	15	2.9	43.5	ASP	random	n.p.	78	73	77	74	−2.6
Molmen-Hansen et al. [96]	♀ + ♂	31	52.5	12	3	36	oscillometer	random	n.p.	73	69.5	73.4	73.7	−5.2
Moreau et al. [97]	♀	15	53	24	6	144	n.p.	random	n.p.	77	75	77	76	−1.3
Morgan et al. [98]	♀ + ♂	14	57.4	15	7	105	ECG	random	n.p.	75.64	71.21	71.6	72.13	−6.5
Nakamura et al. [18]	♀ + ♂	8	22.6	8	3	24	ECG	random	n.p.	78.7	73.4	71	70	−5.4
Nemoto et al. [99]	♀	31	64	22	4	88	ECG	random	n.p.	81	78	79	77	−1.2
Nemoto et al. [99]	♂	11	67	22	4	88	ECG	random	n.p.	75	74	80	77	2.5
Norris et al. [100]	♀ + ♂	14	16.7	10	2	20	n.p.	n.p.	n.p.	78.86	63.71	75.37	77.62	−21.6
Nualnim et al. [101]	♀ + ♂	24	58	12	7	84	ECG	random	n.p.	62	58	63	59	−0.1
Nybo et al. [102]	♂	8	37	12	2	24	ECG	n.p.	n.p.	55	52	60	61	−7.0
Ortega et al. [103]	♀ + ♂	7	24.8	2	5.5	11	ECG	random	n.p.	69	60	62	60	−10.1
Palmer [104]	♀	16	37.4	8	n.p.	n.c.	ASP	random	n.p.	74	72.2	71	72.7	−4.7
Patterson et al. [105]	♀	14	34.9	8	3	24	ECG	random	n.p.	74	66	72	72	−10.8
Pollock et al. [106]	♂	15	48.9	20	4	80	ECG	n.p.	n.p.	65	61.7	73.3	72.4	−3.9
Pollock et al. [107]	♂	9	38	20	3	60	ECG	random	n.p.	67.3	60	68	65.9	−8.0
Pollock et al. [108]	♂	22	55	20	3	60	ECG	n.p.	n.p.	62.9	56.1	67.6	65.6	−8.1
Racil et al. [109]	♀	14.20	17	12	3	36	ECG	random	n.p.	70	67	70	70	−4.3
Ray und Carter [110]	♀ + ♂	14	25	8	4	32	ECG	random	n.p.	66	64	76	75	−1.7
Richter et al. [111]	♂	10	45	9	3	27	ECG	random	n.p.	77	76.4	84	82	1.6
Ruoti et al. [112]	♀ + ♂	12	65	12	3	36	ECG	random	n.p.	72.21	67.12	71.32	72.32	−8.3
Sakai et al. [113]	♀ + ♂	19	56	4	3	12	ASP	random	n.p.	74	74	75	76	−1.3
Sakuragi u. Sugiama [114]	♀	8	19.4	4	6	24	ECG	random	n.p.	65.5	61.2	64	63.9	−6.4
Schmidt et al. [115]	♂	9	68	8	1.5	12	ASP	random	n.p.	64	56	61	59	−9.5
Seals et al. [116]	♀ + ♂	10	62	30.3	3.6	1098	ECG	n.p.	n.p.	68	67	62	66	−7.4
Seals u. Reiling [117]	♀ + ♂	9	63	6	3.6	21.6	n.p.	n.p.	n.p.	83	82	76	79	−5.0
Serwe et al. [118]	♀	20	37.1	8	5	40	n.p.	random	n.p.	68.4	68.6	72.7	72.9	0.0
Shiotani et al. [119]	♀ + ♂	16	young adults	9	3	27	ECG	random	n.p.	73.7	69.5	75	74.7	−5.3
Silie et al. [120]	♀	17	19.8	12	5	60	ECG	random	n.p.	82.3	78.7	82	81.8	−4.1
Spalding et al. [121]	♀ + ♂	15	22.2	6	4	24	ECG	random	n.p.	72.7	71.5	75.5	75.5	−1.7
Stefanick et al. [122]	♀ + ♂	43	30–64	52	3	156	n.p.	random	n.p.	67	66.2	67	68.1	−2.8
Stefanick et al. [122]	♀ + ♂	43	30–64	52	3	156	n.p.	random	n.p.	67	63.2	67	65.4	−3.4
Sugawara et al. [123]	♀	11	59	8	4.5	36	ECG	random	n.p.	59.5	58.5	65.5	64	.6
Suter et al. [124]	♂	39	38.8	17	4	68	ECG	random	n.p.	64.7	61.2	60.7	59.4	−3.3
Tanabe et al. [125]	♀ + ♂	21	50.9	10	3	30	n.p.	n. a	n.p.	75.7	73.2	69.1	70.6	−5.4
Tanaka et al. [126]	♀ + ♂	11	62	10	3	30	n.p.	n. a	n.p.	70	68	66	66	−2.9
Tanaka et al. [127,128,129]	♀ + ♂	12	47	10	3	30	palpation	n. a	n.p.	81	71	76	75	−11.2
Tsai et al. [130,131]	♀ + ♂	12	49.6	12	3	36	n.p.	n.p.	n.p.	83.8	79.2	80.4	79.7	−4.7
Tsai et al. [130,131]	♀ + ♂	22	45.5	12	3	36	n.p.	n.p.	n.p.	79	82.8	84.8	91	−2.3
Tsai et al. [132]	♀ + ♂	37	48.8	10	3	30	n.p.	random	n.p.	76.8	74.6	76.6	78.8	−5.6
Tully et al. [133]	♀ + ♂	42	46.37	12	3	36	ASP	random	n.p.	72	69.05	75	73.9	−2.7
Turner et al. [134]	♀ + ♂	11	65.2	30	4	120	n.p.	n.p.	n.p.	63.4	57.8	68	63	−1.6
Urata et al. [135]	♀ + ♂	10	51.4	10	3	30	n.p.	random	n.p.	74	72.1	73.3	71.2	.3
Vance et al. [136]	♂	19	n.p.	16	n.p.	n. c.	n.p.	n.p.	n.p.	76.71	67.6	73.18	76.05	−5.2
Wanderley et al. [137]	♀ + ♂	20	69.9	34	3	102	ECG	random	n.p.	67.5	64.1	68.4	65.5	−0.8
Whitehurst & Menendez [138]	♀	18	69	8	3	24	ECG	n.p.	n.p.	72.9	68.3	72.9	73.3	−6.8
Winter et al. [139]	♀ + ♂	24	31	10	3	30	ECG	ENV	n.p.	75	70	79	77	−4.2
Wolfe et al. [140]	♀	12	19–22	11	4	44	n.p.	n.p.	n.p.	68	68	67	71	−5.6
Wood et al. [141]	♂	48	45.3	52	4	208	ECG	random	n.p.	66.6	57.4	71.1	68.7	−10.8
Wood et al. [142]	♀ + ♂	11	69.1	12	3	36	ECG	n.p.	n.p.	67.8	62.2	64.1	64.8	−9.3
Wynne et al. [143]	♀	13	23.2	10	3	30	n.p.	n.p.	n.p.	76	68	70	67	−6.5
Yamamoto et al. [144]	♂	7	21	6	4	24	ECG	n.p.	n.p.	68.1	53.2	67.5	66.2	−20.3
Yoshizawa et al. [145]	♀	12	47	12	2	24	n.p.	random	n.p.	67	59	61	62	−13.4
**Combined endurance and strength training**														
Anek et al. [27]	♀	15	40.26	16	3	48	ECG	random	n.p.	79.4	75.2	79.13	79.56	−5.8
Byrne u. Wilmore [146]	♀	10	35.9	20	3	60	ECG	random	n.p.	66.5	59.3	63	62.2	−9.7
Cortez-Cooper et al. [147]	♀ + ♂	12	51	13	2	26	n.p.	random	n.p.	62	59	65	65	−4.8
Delecluse et al. [47]	♂	18	63.8	20	2.5	50	ECG	random	n.p.	76.2	71.4	73.3	73.9	−7.1
Deley et al. [148]	♀ + ♂	24	77.2	52	3	156	ECG	random	n.p.	73	75.5	74.3	70.1	9.6
Figueroa et al. [149]	♀	12	54	12	3	36	ECG	random	n.p.	66	62	68	67	−4.7
Frye et al. [150]	♀ + ♂	28	69.2	12	3	36	n.p.	random	n.p.	66.8	70	68.7	67.9	6.0
Karavirta et al. [75]	♂	29	55.6	21	2	42	ECG	random	n.p.	58	57	54	53	0.1
Karavirta et al. [76]	♀	21	49	21	2	42	ECG	random	n.p.	62	62	65	62	4.8
Masroor et al. [151]	♀	15	39.67	4	5	20	ECG	random	n.p.	75.5	71.2	80.3	83.2	−9.0
Ohkubo et al. [152]	♀ + ♂	22	60–81	25	2	50	ASP	random	n.p.	62.6	60.2	66.2	64	−0.5
Stewart et al. [153,154]	♀ + ♂	51	63	26	3	78	ASP	random	n.p.	69.8	65.9	71.9	69.7	−2.6
Svendsen et al. [155]	♀	48	53.8	12	3	36	n.p.	random	n.p.	73	64	74	69	−6.0
Tsuda et al. [156]	♂	8	46.2	6	2	12	ECG	random	n.p.	69	68	72	70	1.4
Wood et al. [142]	♀ + ♂	9	66.1	12	3	36	ECG	n.p.	n.p.	79.8	73.7	64.1	64.8	−8.6
**Strength training**														
Badrov et al. [157]	♀	11	27	8	5	40	ECG	random	n.p.	64	64	66	65	1.5
Baross et al. [158]	♂	10	55	8	3	24	ECG	random	n.p.	71	66	69	68.2	−6.0
Beck et al. [32,33]	♀ + ♂	15	21.1	8	3	24	n.p.	random	n.p.	63	61	60	58	0.2
Byrne u. Wilmore [146]	♀	9	39.1	20	4	80	ECG	random	n.p.	63.4	60.5	63	62.2	−3.3
Carter et al. [159]	♀ + ♂	12	21	8	3	24	ECG	non random	n.p.	65	63	61	67	−11.8
Cononie et al. [45]	♀ + ♂	12	72	26	3	78	ECG	random	n.p.	61	65	68	67	8.1
Cononie et al. [45]	♀ + ♂	6	72	26	3	78	ECG	random	n.p.	65	69	66	65	7.8
Cortez-Cooper et al. [147]	♀ + ♂	13	52	13	3	39	n.p.	random	n.p.	60	61	65	65	1.7
Fripp et al. [160]	♂	14	15.2	9	3	27	n.p.	n.p.	n.p.	76	78	79	79	2.6
Gelecek et al. [161]	♀	24	54.33	12	3	36	palpation	random	single blinded	80.08	78.41	77.76	80.47	−5.4
Gerage et al. [162]	♀	15	65.5	12	3	36	ECG	random	n.p.	73.86	71.89	78.37	81.14	−6.0
Giannaki et al. [163]	♂	20	16	8	2	16	ECG	random	n.p.	78.8	77.6	76.8	78.5	−3.7
Gorjao et al. [164]	♀	10	66	8	3	24	ECG	random	n.p.	75.7	77.4	76.6	74.7	4.8
Harris und Holly [165]	♂	10	32.7	9	3	27	ECG	random	n.p.	75.7	76.5	73.9	72.3	3.3
Ibrahim et al. [166]	♂	12	21	12	3	36	ASP	random	n.p.	71.7	71.2	72.9	72.6	−0.3
Ibrahim et al. [166]	♂	10	22	12	3	36	ASP	random	n.p.	72.4	68.3	72.2	68.5	−0.6
Kanegusuku et al. [167]	♀ + ♂	13	63	16	2	32	ECG	random	n.p.	83	87	81	78	8.9
Karavirta et al. [75]	♂	25	55.6	21	2	42	ECG	random	n.p.	59	59	54	53	1.9
Karavirta et al. [76]	♀	26	52	21	2	42	ECG	random	n.p.	62	61	65	62	3.1
Lovell et al. [168]	♂	12	71.4	16	3	48	n.p.	random	n.p.	69	68	68	66	1.5
Marinda et al. [169]	♀	25	66.12	8	3	24	n.p.	random	n.p.	68.8	73.2	62.48	74.92	−1.3
Menezes-Cabral et al. [91]	♀	51	64.8	17	4	68	n.p.	n.p.	n.p.	75.7	72	77.6	78.7	−6.2
Menezes-Cabral et al. [91]	♂	53	64.5	17	4	68	n.p.	n.p.	n.p.	82.6	77.1	87	87.9	−7.6
Millar et al. [170]	♀ + ♂	13	65	8	3	24	ECG	non random	n.p.	58	56	62	63	−5.0
Miyachi et al. [171]	♂	14	22	17	3	51	n.p.	random	n.p.	55	54	57	56	−0.1
Mogharnesi et al. [94]	♂	12	13.71	8	3	24	auscultation	random	n.p.	76.5	70.33	74.6	75.6	−9.3
Nybo et al. [102]	♂	8	36	12	2	24	ASP	n.p.	n.p.	57	56	60	61	−3.4
Okamoto et al. [172]	♀	10	19.1	8	3	24	n.p.	random	n.p.	68.4	67.1	67.6	62.4	6.3
Okamoto et al. [173]	♀ + ♂	13	18.5	10	2	20	n.p.	random	n.p.	66	66	63	62	1.6
Schmidt et al. [115]	♂	9	69.1	12	1.9	22.8	ASP	random	n.p.	63	61	61	59	0.1
Shaw et al. [174]	♀	19	60.44	6	2	12	auscultation	random	n.p.	69.05	63.8	71.26	71	−7.3
Spalding et al. [121]	♀ + ♂	15	22.2	6	4	24	pulse monitor	random	n.p.	72.9	73	75.5	75.5	0.1
Stiller-Moldovan et al. [175]	♀ + ♂	11	60	8	3	24	pulse monitor	random	n.p.	71.3	71	66.2	64.4	2.4
Taylor et al. [176]	♀ + ♂	9	69.3	10	3	30	ECG	random	n.p.	70	68	70	76	−10.5
Terra et al. [177]	♀	20	66.8	12	3	36	ASP	non random	n.p.	72.2	72.7	74.1	74	0.8
Thomas et al. [178]	♀ + ♂	65	69.1	12	3	36	ASP	random	n.p.	67	65	67	65	0.0
Vinvent et al. [179]	♀ + ♂	24	66.6	24	3	72	auscultation	random	n.p.	82	80	77	84	−10.6
Wanderley et al. [137]	♀ + ♂	11	67.3	34	3	102	ECG	random	n.p.	62.2	60.3	68.4	65.5	1.2
Wiley et al. [21]	n.p.	10	20–35	8	3	24	ECG	random	n.p.	78	76	77	82	−8.5
Wilmore et al. [180]	♀	12	n.p.	10	3	30	ECG	random	n.p.	65.4	59.4	61.5	59.6	−6.3
Wilmore et al. [180]	♂	16	n.p.	10	3	30	ECG	random	n.p.	63.6	58.4	61.8	55.3	2.6
Wood et al. [142]	♀ + ♂	10	69.8	12	3	36	ECG	n.p.	n.p.	67.3	63.5	64.1	64.8	−6.7
Yoshizawa et al. [145]	♀	11	47	12	2	24	n.p.	random	n.p.	67	63	61	62	−7.5
**Unspecified sports activities**														
Edwards et al. [181]	♀ + ♂	25	46.5	12	5	60	ECG	random	n.p.	77.2	73.3	75.4	78.5	−8.8
García-Ortiz et al. [182]	♀ + ♂	1456	51.47	52	4	208	n.p.	random	n.p.	75.8	75.42	75.27	74.89	0.0
**Additional school sports**														
Hansen et al. [183]	♀	17	9–11	8	3	24	ECG	random	n.p.	91.8	85.9	96	88	2.1
Nogueira et al. [184]	♀	71	10.6	35	3	105	auscultation	random	n.p.	71.8	66.6	67.2	66	−5.6
Rautela et al. [185]	♀	30	n.p.	6	6	36	ECG	random	n.p.	80.9	80.73	81.1	81	−0.1
Walther et al. [186]	♀ + ♂	141	11	104	3	312	n.p.	ENV	n.p.	103	98.3	102	98	−0.7
Wong et al. [187]	♂	12	13.75	12	2	24	ECG	random	n.p.	78	71	76	76	−9.0
**Qigong**														
Lee et al. [188]	♀ + ♂	29	55.8	10	3	30	ASP	random	n.p.	73.82	72.27	73.55	76.2	−5.5
Li et al. [189]	♀ + ♂	101	20.63	12	5	60	n.p.	PLAN	n.p.	83.61	82.61	80.49	82.22	−3.3
Sousa et al. [190]	♀	8	11.5	7	7	49	n.p.	non random	n.p.	107	96	81	86	−15.5
**Tai chi**														
Frye et al. [150]	♀ + ♂	23	69.2	12	3	36	n.p.	random	n.p.	70.95	70.77	68.7	67.9	0.9
Logghe et al. [191]	♀ + ♂	138	77.5	13	2	26	n.p.	random	n.p.	71	68.3	70.6	67.8	0.2
Nguyen und Kruse [192]	♀ + ♂	48	69.23	26	2	52	n.p.	random	n.p.	84.48	76.72	83.08	82.86	−8.9
Thomas et al. [178]	♀ + ♂	64	68.9	52	3	156	ASP	random	n.p.	70	67	67	65	−1.3
Tsai et al. [193]	♀ + ♂	37	51.6	12	3	36	ECG	random	n.p.	77.8	75.8	78.2	76.6	−0.5
**Yoga**														
Bezerra et al. [194]	♀	18	63.1	12	3	36	oximeter	random	n.p.	76.39	74.61	77.28	79.78	−5.4
Blumenthal et al. [35,36]	♀ + ♂	32	67	17	2	34	ECG	random	n.p.	75	70	70	69	−5.3
Cheema et al. [195]	♀ + ♂	18	37	10	3	30	ECG	random	n.p.	62	65	68	67	6.4
Cohen et al. [196]	♀ + ♂	46	48.2	6	3	18	n.p.	random	n.p.	70	68	69	67	0.0
Hewett et al. [197]	♀ + ♂	29	38.2	16	4	64	ECG	random	n.p.	64.1	62.9	65.4	62.2	3.2
Kanojia et al. [198]	♀	25	18.6	13	6	78	ECG	random	n.p.	79.84	72.96	75.72	81.92	−15.5
Kim et al. [199]	♀	16	45.7	6	5	30	pulse monitor	random	n.p.	65.9	65.9	65.8	66.1	−0.5
Krishna et al. [200]	♀ + ♂	44	49.34	12	1	12	ECG	random	n.p.	96.36	81.48	96.33	92.04	−11.5
Lau et al. [201]	♀	53	51.64	12	3	36	ECG	non random	n.p.	69.11	66.81	70.61	71.39	−4.4
Lau et al. [201]	♂	34	53.68	12	3	36	ECG	non random	n.p.	67.53	66.32	66.77	68.73	−4.6
Madanmohan et al. [202]	♀ + ♂	23	17–20	6	6	36	ASP	n.p.	n.p.	70	67	75	73	−1.7
McCaffrey et al. [203]	♀ + ♂	27	56.7	8	3	24	n.p.	random	n.p.	85.59	73.74	80.85	85.07	−18.1
Mehrotra et al. [204]	♀	36	18–21	13	7	91	ECG	non random	n.p.	86.84	72.97	81.68	82.35	−16.7
Mehrotra et al. [204]	♂	36	18–21	13	7	91	ECG	non random	n.p.	77.08	70.14	80.59	79.76	−8.1
Murugesan et al. [205]	♀ + ♂	11	35–65	11	6	66	palpation	random	n.p.	92.61	64.62	90.53	88.25	−28.4
Ray et al. [206]	♀	5	22.6	22	3	66	auscultation	random	n.p.	77.6	70.2	80.2	73.2	−0.9
Ray et al. [206]	♂	23	23.6	22	3	66	auscultation	random	n.p.	74.91	73.61	74.85	75.45	−2.5
Sieverdes et al. [207]	♀ + ♂	14	12.1	13	2.5	32.5	ASP	random	n.p.	74.8	72.8	78.5	82.64	−7.5
Tew et al. [208]	♀	25	73.8	12	0.8	9.6	n.p.	random	n.p.	79	74	80	80	−6.3
Thiyagarajan et al. [209]	♀ + ♂	51	44.08	12	3	36	n.p.	c.r.	n.p.	75	72	77	74	−0.1
Udupa et al. [210]	♂	31	14.5	13	5	65	ASP	random	n.p.	73.58	67.42	76.15	74.64	−6.5

Notes: ♀ + ♂ = males and females; ♀ = females only; ♂ = males only; adol. = adolescents; ASP = automatic sphygmanometer; CG = control group; c.r. = cluster randomization; ECG = electrocardiography; ENV = choosing an envelope which contained the allocation towards the treatment or control group; IG = intervention group; min = minute(s); IVRS = interactive voice response system; *N* = number of participants; n.c. = not calculable; n.p. = not presented; PLAN = PLAN sentences of the statistical software SAS9.1; PMP = postmenopausal; rel. = relative; RHR = resting heart rate; wk = week(s); y = year(s); * mean, median, or range.

**Table 2:** Effects of regular exercise/sports on RHR according to type of sports/exercise.

Types of Sports/Exercise Participants’ Sex, Number of Comparisons (c), Participants in the Intervention (*N*_IG_), and Control Groups (*N*_CG_)	Meta-Analytic SMD +95%-CI +95%-HK-CI	Heterogeneity	IG		CG		Correlation between Baseline RHR and Change of RHR After Intervention ^*^
			Baseline RHR (M(SE))	Final RHR: rel. Change (M(SE))	Baseline RHR (M(SE))	Final RHR: rel. Change (M(SE))	
**All types of sports**							
all *c* =215, *N*_IG_ = 6763, *N*_CG_ = 6189	SMD = −0.29 (95%-CI: −0.35–−0.24, *p* < 0.001) (95%-HK-CI: −0.35–−0.24, *p* < 0.001)	*Q* = 381.06, *p* < 0.001, *I*^2^ = 43.8%	72.4 (0.5)	68.7 (0.5) −5.2 (0.4)%	72.3 (0.6)	71.9 (0.6) −0.5 (0.2)%	−0.36 (95%-CI: −0.47–−0.24, *p* < 0.001)
females only *c* = 65, *N*_IG_ = 1366, *N*_CG_ = 1268	SMD = −0.35 (95%-CI: −0.43–−0.26, *p* < 0.001) (95%-HK-CI: −0.43–−0.26, *p* < 0.001)	*Q* = 67.17, *p* = 0.305, *I*^2^ = 7.7%	73.2 (0.8)	69.5 (0.9) −5.1 (0.6)%	73.2 (1.0)	72.9 (0.9) −0.3 (0.2)%	−0.32 (95%-CI: −0.52–−0.08, *p* = 0.009)
males only *c* = 58, *N*_IG_ = 1208, *N*_CG_ = 1036	SMD = −0.40 (95%-CI: −0.51–−0.29, *p* < 0.001) (95%-HK-CI: −0.52–−0.28, *p* < 0.001)	*Q* = 79.35, *p* = 0.018, *I*^2^ = 30.7%	70.3 (1.2)	65.4 (1.1) −7.0 (0.6)%	70.5 (1.6)	69.9 (1.6) −0.6 (0.4)%	−0.22 (95%-CI: −0.46–0.04, *p* = 0.094)
females and males *c* = 92, *N*_IG_ = 4189, *N*_CG_ = 3885	SMD = −0.21 (95%-CI: −0.29–−0.13, *p* < 0.001) (95%-HK-CI: −0.30–−0.12, *p* < 0.001)	*Q* = 185.24, *p* < 0.001, *I*^2^ = 50.3%	73.1 (0.7)	70.0 (0.7) −4.3 (0.6)%	72.7 (0.9)	72.3 (0.9) −0.6 (0.4)%	−0.49 (95%-CI: −0.63–−0.32, *p* < 0.001)
**Endurance training**							
all *c* = 121, *N*_IG_ = 2924, *N*_CG_ = 2533	SMD = −0.33 (95%-CI: −0.40–−0.26, *p* < 0.001) (95%-HK-CI: −0.40–−0.26, *p* < 0.001)	*Q* = 157.16, *p* = 0.013, *I*^2^ = 23.6%	72.4 (0.6)	68.0 (0.7) −6.0 (0.4)%	72.3 (0.8)	71.9 (0.8) −0.1 (0.3)%	−0.19 (95%-CI: −0.36–−0.01, *p* = 0.042)
females only *c* = 35, *N*_IG_ = 698, *N*_CG_ = 620	SMD = −0.38 (95%-CI: −0.49–−0.26, *p* < 0.001) (95%-HK-CI: −0.50–−0.26, *p* < 0.001)	*Q* = 37.06, *p* = 0.330, *I*^2^ = 8.3%	73.6 (1.2)	69.8 (1.1) −5.2 (0.6)%	73.4 (1.5)	73.3 (1.3) −0.2 (0.4)%	−0.26 (95%-CI: −0.55–0.08, *p* = 0.132)
males only *c* = 36, *N*_IG_ = 792, *N*_CG_ = 656	SMD = −0.49 (95%-CI: −0.63–−0.35, *p* < 0.001) (95%-HK-CI: −0.64–−0.34, *p* < 0.001)	*Q* = 45.18, *p* = 0.077, *I*^2^ = 27.0%	70.3 (1.6)	64.0 (1.6) −9.0 (0.7)%	70.8 (2.1)	70.3 (2.2) 0.0 (0.6)%	−0.18 (95%-CI: −0.49–0.17, *p* = 0.320)
females and males *c* = 50, *N*_IG_ = 1434, *N*_CG_ = 1277	SMD = −0.19 (95%-CI: −0.28–−0.11, *p* < 0.001) (95%-HK-CI: −0.28–−0.10, *p* < 0.001)	*Q* = 53.85, *p* = 0.294, *I*^2^ = 9.0%	72.8 (0.7)	69.4 (0.8) −4.6 (0.7) %	72.4 (0.8)	71.9 (0.9) −0.0 (0.5)%	−0.33 (95%-CI: −0.56–−0.05, *p* = 0.022)
**Strength training**							
all *c* = 43, *N*_IG_ = 720, *N*_CG_ = 689	SMD = −0.18 (95%-CI: −0.32–−0.04, *p* = 0.011) (95%-HK-CI: −0.32–−0.04, *p* = 0.014)	*Q* = 64.98, *p* = 0.013, *I*^2^ = 35.4%	69.1 (1.2)	67.8 (1.2) −2.5 (0.7)%	68.9 (1.3)	69.0 (1.4) 0.1 (0.8)%	−0.04 (95%-CI: −0.34–0.27, *p* = 0.817)
females only *c* = 13, *N*_IG_ = 243, *N*_CG_ = 229	SMD = −0.26 (95%-CI: −0.44–−0.08, *p* = 0.005) (95%-HK-CI: −0.44–−0.09, *p* = 0.007)	*Q* = 9.07, *p* = 0.697, *I*^2^ = 0%	69.7 (1.6)	68.1 (1.8) −3.7 (1.0)%	69.4 (1.9)	70.0 (2.1) 1.3 (1.6)%	0.19 (95%-CI: −0.43–0.69, *p* = 0.565)
males only *c* = 14, *N*_IG_ = 225, *N*_CG_ = 214	SMD = −0.23 (95%-CI: −0.48–0.03, *p* = 0.081) (95%-HK-CI: −0.48–0.03, *p* = 0.075)	*Q* = 21.39, *p* = 0.066, *I*^2^ = 39.2%	69.4 (2.6)	67.3 (2.4) −4.2 (0.9)%	69.1 (3.1)	68.1 (3.4) −1.7 (1.0)%	−0.21 (95%-CI: −0.67–0.36, *p* = 0.465)
females and males *c* = 16, *N*_IG_ = 252, *N*_CG_ = 246	SMD = −0.06 (95%-CI: −0.34–0.23, *p* = 0.704) (95%-HK-CI: −0.40–0.29, *p* = 0.737)	*Q* = 28.95, *p* = 0.011, *I*^2^ = 51.6%	67.6 (1.9)	67.4 (2.1) −0.1 (1.0)%	67.6 (1.5)	68.0 (1.8) 0.5 (1.9)%	0.15 (95%-CI: −0.39–0.61, *p* = 0.597)
**Combined endurance and strength training**							
all *c* = 15, *N*_IG_ = 322, *N*_CG_ = 279	SMD = −0.18 (95%-CI: −0.35–−0.01, *p* = 0.041) (95%-HK-CI: −0.37–0.01, *p* = 0.061)	*Q* = 15.30, *p* = 0.358, *I*^2^ = 8.5%	69.1 (1.5)	66.3 (1.8) −4.8 (0.8)%	69.2 (2.0)	68.1 (2.4) −1.5 (0.8)%	−0.35 (95%-CI: −0.73–0.19, *p* = 0.195)
females only *c* = 6, *N*_IG_ = 121, *N*_CG_ = 117	SMD = −0.38 (95%-CI: −0.72–−0.03, *p* = 0.031) (95%-HK-CI: −0.83–0.08, *p* = 0.086)	*Q* = 7.70, *p* = 0.174, *I*^2^ = 35.1%	70.5 (3.4)	65.7 (3.2) −5.9 (1.5)%	71.6 (3.4)	70.6 (3.8) −1.4 (1.5)%	−0.37 (95%-CI: −0.91–0.63, *p* = 0.475)
males only *c* = 3, *N*_IG_ = 55, *N*_CG_ = 37	SMD = −0.10 (95%-CI: −0.52–0.32, *p* = 0.654) (95%-HK-CI: −0.61–0.41, *p* = 0.503)	*Q* = 0.61, *p* = 0.736, *I*^2^ = 0.0%	67.5 (6.0)	65.3 (4.7) −2.7 (2.5)%	66.1 (7.5)	65.5 (6.9) −1.4 (2.7)%	
females and males *c* = 6, *N*_IG_ = 146, *N*_CG_ = 125	SMD = −0.06 (95%-CI: −0.29–0.19, *p* = 0.679) (95%-HK-CI: −0.32–0.22, *p* = 0.653)	*Q* = 3.76, *p* = 0.584, *I*^2^ = 0%	68.4 (2.0)	67.3 (2.5) −4.3 (1.3)%	68.4 (1.8)	67.4 (1.2) −1.5%	−0.18 (95%-CI: −0.86–0.74, *p* = 0.738)
**Additional school sports**							
all *c* = 5, *N*_IG_ = 271, *N*_CG_ = 216	SMD = −0.26 (95%-CI: −0.63–0.10, *p* = 0.154) (95%-HK-CI: −0.92–0.39, *p* = 0.328)	*Q* = 12.19, *p* = 0.016, *I*^2^ = 67.2%	84.8 (3.9)	80.4 (4.1) −5.2 (2.3)%	84.3 (4.8)	81.6 (4.4) −1.4 (1.0)%	−0.09 (95%-CI: −0.90–0.86, *p* = 0.883)
**Unspecified sports activities**							
all *c* = 2, *N*_IG_ = 1481, *N*_CG_ = 1414	SMD = −0.13 (95%-CI: −0.52–0.26, *p* = 0.523)	*Q* = 2.3, *p* = 0.130, *I*^2^ = 56.4%	75.8 (0.3)	75.1 (0.7) −0.9 (1.0)%	75.4 (0.3)	76.3 (1.8) 0.7 (2.2)%	
**Yoga**							
all *c* = 21, *N*_IG_ = 597, *N*_CG_ = 589	SMD = −0.37 (95%-CI: −0.58–−0.16, *p* < 0.001) (95%-HK-CI: −0.60–−0.14, *p* = 0.003)	*Q* = 59.32, *p* < 0.001, *I*^2^ = 66.3%	76.1 (2.5)	70.4 (1.3) −7.2 (1.6)%	76.7 (2.4)	75.9 (2.1) −2.0 (0.7)%	−0.87 (95%-CI: −0.94–−0.69, *p* < 0.001)
females only *c* = 7, *N*_IG_ = 178, *N*_CG_ = 181	SMD = −0.44 (95%-CI: −0.76–−0.13, *p* = 0.006) (95%-HK-CI: −0.84–−0.04, *p* = 0.036)	*Q* = 8.36, *p* = 0.138, *I*^2^ = 40.2%	76.9 (3.6)	71.4 (2.6) −7.2 (2.3)%	77.1 (3.4)	76.2 (3.2) −1.7 (1.6)%	−0.89 (95%-CI: −0.99–−0.28, *p* = 0.018)
males only *c* = 4, *N*_IG_ = 124, *N*_CG_ = 117	SMD = −0.24 (95%-CI: −0.50–0.01, *p* = 0.063) (95% HCI: −0.38–−0.10, *p* = 0.013)	*Q* = 0.36, *p* = 0.949, *I*^2^ = 0%	73.2 (2.3)	69.6 (1.7) −4.8 (2.1)%	74.4 (3.0)	74.5 (2.7) 0.7 (2.0)%	
females and males *c* = 10, *N*_IG_ = 295, *N*_CG_ = 291	SMD = −0.41 (95%-CI: −0.77–−0.05, *p* = 0.028) (95%-HK-CI: −0.86–0.04, *p* = 0.069)	*Q* = 49.28, *p* < 0.001, *I*^2^ = 79.7%	76.8 (4.4)	70.2 (2.1) −7.9 (2.7)%	77.3 (4.0)	76.3 (3.7) −2.6 (1.0)%	−0.87 (95%-CI: −0.97–−0.58, *p* < 0.001)
**Tai chi**							
all *c* = 5, *N*_IG_ = 310, *N*_CG_ = 317	SMD = −0.26 (95%-CI: −0.68–0.16, *p* = 0.228) (95% HCI: −0.89–0.38, *p* = 0.327)	*Q* = 24.03, *p* < 0.001, *I*^2^ = 83.4%	74.9 (3.2)	71.9 (2.1) −4.7 (1.8)%	73.6 (3.5)	72.1 (4.2) −1.1 (0.8)%	−0.78 (95%-CI: −0.99–0.31, *p* = 0.115)
**Qigong**							
all *c* = 3, *N*_IG_ = 138, *N*_CG_ = 142	SMD = −0.23 (95%-CI: −0.47–0.00, *p* = 0.054)	*Q* = 1.66, *p* = 0.437, *I*^2^ = 0%	84.6 (5.1)	81.6 (4.9) −1.9 (1.5)%	78.1 (2.7)	81.2 (2.6) 3.0 (1.7)%	
**All Asian sports**							
all *c* = 29, *N*_IG_ = 1045, *N*_CG_ = 1038	SMD = −0.34 (95%-CI: −0.50–−0.18, *p* < 0.001) (95%-HK-CI: −0.52–−0.16, *p* < 0.001)	*Q* = 87.81, *p* < 0.001, *I*^2^ = 68.1%	76.8 (1.8)	71.6 (1.0) −6.2 (1.2)%	76.3 (1.7)	75.8 (1.6) −1.3 (0.6)%	−0.75 (95%-CI: −0.88–−0.54, *p* < 0.001)
females and males *c* = 17, *N*_IG_ = 735, *N*_CG_ = 732	SMD = −0.35 (95%-CI: −0.57–−0.12, *p* = 0.002) (95%-HK-CI: −0.61–−0.08, *p* = 0.013)	*Q* = 76.90, *p* < 0.001, *I*^2^ = 76.6%	77.5 (2.4)	72.1 (1.3) −6.2 (1.6)%	76.5 (2.3)	76.0 (2.1) −1.3 (0.7)%	−0.74 (95%-CI: −0.89–−0.43, *p* < 0.001)

* At least 5 trials; *c* = number of comparisons; CI = confidence interval; HK-CI = Hartung–Knapp confidence interval; *I*^2^ = Higgins–Thompson *I*^2^; M = mean; *N*_CG_ = participants in the control groups; *N*_IG_ = participants in the intervention groups (exercising participants); *Q* = Cochran’s *Q*; rel. change = relative change as compared to baseline; RHR = resting heart rate; s = studies (publications); SE = standard error; SMD = standardized mean difference.

## References

[1] Tesch-Römer C., Wurm S., Robert Koch Institut (2009). Wer sind die Alten? Theoretische Positionen zum Alter und Altern. Gesundheit und Krankheit im Alter.

[2] Reimers C.D., Knapp G., Reimers A.K. (2012). Does Physical Activity Increase Life Expectancy? A Review of the Literature. J. Aging Res..

[3] Samitz G., Egger M., Zwahlen M. (2011). Domains of physical activity and all-cause mortality: Systematic review and dose-response meta-analysis of cohort studies. Int. J. Epidemiol..

[4] May A.M., Struijk E.A., Fransen H.P., Onland-Moret N.C., de Wit G.A., Boer J.M., van der Schouw Y.T., Hoekstra J., Bueno-de-Mesquita H.B., Peeters P.H. (2015). The impact of a healthy lifestyle on Disability-Adjusted Life Years: A prospective cohort study. BMC Med..

[5] Bronnum-Hansen H., Juel K., Davidsen M., Sorensen J. (2007). Impact of selected risk factors on quality-adjusted life expectancy in Denmark. Scand. J. Public. Health.

[6] Huang G., Shi X., Davis-Brezette J.A., Osness W.H. (2005). Resting heart rate changes after endurance training in older adults: A meta-analysis. Med. Sci. Sports Exerc..

[7] Zheng G., Li S., Huang M., Liu F., Tao J., Chen L. (2015). The effect of Tai Chi training on cardiorespiratory fitness in healthy adults: A systematic review and meta-analysis. PLoS ONE.

[8] Cramer H., Lauche R., Haller H., Steckhan N., Michalsen A., Dobos G. (2014). Effects of yoga on cardiovascular disease risk factors: A systematic review and meta-analysis. Int. J. Cardiol..

[9] Hartaigh B.O., Gill T.M., Shah I., Hughes A.D., Deanfield J.E., Kuh D., Hardy R. (2014). Association between resting heart rate across the life course and all-cause mortality: Longitudinal findings from the Medical Research Council (MRC) National Survey of Health and Development (NSHD). J. Epidemiol. Community Health.

[10] Aune D., Sen A., o’Hartaigh B., Janszky I., Romundstad P.R., Tonstad S., Vatten L.J. (2017). Resting heart rate and the risk of cardiovascular disease, total cancer, and all-cause mortality—A systematic review and dose-response meta-analysis of prospective studies. Nutr. Metab. Cardiovasc. Dis..

[11] Levine H.J. (1997). Rest heart rate and life expectancy. J. Am. Coll. Cardiol..

[12] Custodis F., Reil J.C., Laufs U., Bohm M. (2013). Heart rate: A global target for cardiovascular disease and therapy along the cardiovascular disease continuum. J. Cardiol..

[13] Fagundes J.E., Castro I. (2010). Predictive value of resting heart rate for cardiovascular and all-cause mortality. Arq. Brasil. Cardiol..

[14] Moher D., Liberati A., Tetzlaff J., Altman D.G., Group P. (2009). Preferred reporting items for systematic reviews and meta-analyses: The PRISMA statement. BMJ.

[15] Zou L., SasaKi J.E., Wang H., Xiao Z., Fang Q., Zhang M. (2017). A Systematic Review and Meta-Analysis Baduanjin Qigong for Health Benefits: Randomized Controlled Trials. Evid. Based Complement. Altern. Med..

[16] Reimers C.D., Knapp G., Mooren F., Knapp G., Reimers C.D. (2016). Arterielle Hypertonie. Prävention und Therapie Durch Sport.

[17] Reimers C.D., Knapp G., Mooren F., Knapp G., Reimers C.D. (2016). Dyslipidämien. Prävention und Therapie Durch Sport.

[18] Nakamura Y., Hayashi N., Muraoka I. (1995). Effect of physical training on autonomic nervous system. Butt Phys. Fit. Res. Inst..

[19] Team R.C. (2018). R: A Language and Environment for Statistical Computing.

[20] Hartung J., Knapp G. (2001). On tests of the overall treatment effect in meta-analysis with normally distributed responses. Stat. Med..

[21] Wiley R.L., Dunn C.L., Cox R.H., Hueppchen N.A., Scott M.S. (1992). Isometric exercise training lowers resting blood pressure. Med. Sci. Sports Exerc..

[22] Abadi F.H., Elumalai G., Sankaraval M., Ramli F.A.B.M. (2017). Effects of aqua-aerobic exercise on the cardiovascular fitness and weight loss among obese students. Int. J. Physiother..

[23] Akinpelu A.O. (1990). Responses of the African hypertensive to exercise training: Preliminary observations. J. Hum. Hypertens..

[24] Akwa L.G., Moses M.O., Emikpe A.O., Baffour-Awuah B., Asamoah B., Addai-Mensah O., Annani-Akollor M., Osei F., Appiah E.J. (2017). Lipid profile, cardiorespiratory function and quality of life of postmenopausal women improves with aerobic exercise. J. Hum. Sport Exerc..

[25] Andersen L.J., Randers M.B., Westh K., Martone D., Hansen P.R., Junge A., Dvorak J., Bangsbo J., Krustrup P. (2010). Football as a treatment for hypertension in untrained 30–55-year-old men: A prospective randomized study. Scand. J. Med. Sci. Sports.

[26] Anek A., Bunyaratavej N. (2015). Effects of Circuit Aerobic Step Exercise Program on Musculoskeletal for Prevention of Falling and Enhancement of Postural Balance in Postmenopausal Women. J. Med. Assoc. Thail..

[27] Anek A., Kanungsukasem V., Bunyaratavej N. (2015). Effects of Aerobic Step Combined with Resistance Training on Biochemical Bone Markers, Health-Related Physical Fitness and Balance in Working Women. J. Med. Assoc. Thail..

[28] Apekey T.A., Morris A., Fagbemi S., Griffiths G. (2012). Benefits of moderate-intensity exercise during a calorie-restricted low-fat diet. Health Educ. J..

[29] Aweto H.A., Owoeye O.B., Akinbo S.R., Onabajo A.A. (2012). Effects of dance movement therapy on selected cardiovascular parameters and estimated maximum oxygen consumption in hypertensive patients. Niger. Q. J. Hosp. Med..

[30] Baker G., Gray S.R., Wright A., Fitzsimons C., Nimmo M., Lowry R., Mutrie N., Scottish Physical Activity Research C. (2008). The effect of a pedometer-based community walking intervention “Walking for Wellbeing in the West” on physical activity levels and health outcomes: A 12-week randomized controlled trial. Int. J. Behav. Nutr. Phys. Act..

[31] Baker G., Gray S.R., Wright A., Fitzsimons C., Nimmo M., Lowry R., Mutrie N., Scottish Physical Activity Research C. (2010). Erratum to: The effect of a pedometer-based community walking intervention “Walking for Wellbeing in the West” on physical activity levels and health outcomes: A 12-week randomized controlled trial. Int. J. Behav. Nutr. Phys. Act..

[32] Beck D.T., Casey D.P., Martin J.S., Emerson B.D., Braith R.W. (2013). Exercise training improves endothelial function in young prehypertensives. Exp. Biol. Med..

[33] Beck D.T., Martin J.S., Casey D.P., Braith R.W. (2013). Exercise training reduces peripheral arterial stiffness and myocardial oxygen demand in young prehypertensive subjects. Am. J. Hypertens..

[34] Blum S.M., Sherman A.R., Boileau R.A. (1986). The effects of fitness-type exercise on iron status in adult women. Am. J. Clin. Nutr..

[35] Blumenthal J.A., Emery C.F., Madden D.J., Coleman R.E., Riddle M.W., Schniebolk S., Cobb F.R., Sullivan M.J., Higginbotham M.B. (1991). Effects of exercise training on cardiorespiratory function in men and women older than 60 years of age. Am. J. Cardiol..

[36] Blumenthal J.A., Emery C.F., Madden D.J., Schniebolk S., Walsh-Riddle M., George L.K., McKee D.C., Higginbotham M.B., Cobb F.R., Coleman R.E. (1991). Long-term effects of exercise on psychological functioning in older men and women. J. Gerontol..

[37] Bocalini D.S., Serra A.J., Murad N., Levy R.F. (2008). Water- versus land-based exercise effects on physical fitness in older women. Geriatr. Gerontol. Int..

[38] Braith R.W., Pollock M.L., Lowenthal D.T., Graves J.E., Limacher M.C. (1994). Moderate- and high-intensity exercise lowers blood pressure in normotensive subjects 60 to 79 years of age. Am. J. Cardiol..

[39] Boutcher S.H., Stein P. (1995). Association between heart rate variability and training response in sedentary middle-aged men. Eur. J. Appl. Physiol. Occup. Physiol..

[40] Broman G., Quintana M., Lindberg T., Jansson E., Kaijser L. (2006). High intensity deep water training can improve aerobic power in elderly women. Eur. J. Appl. Physiol..

[41] Carroll J.F., Convertino V.A., Wood C.E., Graves J.E., Lowenthal D.T., Pollock M.L. (1995). Effect of training on blood volume and plasma hormone concentrations in the elderly. Med. Sci. Sports Exerc..

[42] Ciolac E.G., Bocchi E.A., Greve J.M., Guimaraes G.V. (2011). Heart rate response to exercise and cardiorespiratory fitness of young women at high familial risk for hypertension: Effects of interval vs continuous training. Eur. J. Cardiovasc. Prev. Rehabil..

[43] Connolly L.J., Scott S., Mohr M., Ermidis G., Julian R., Bangsbo J., Jackman S.R., Bowtell J.L., Davies R.C., Hopkins S.J. (2014). Effects of small-volume soccer and vibration training on body composition, aerobic fitness, and muscular PCr kinetics for inactive women aged 20–45. J. Sport. Health Sci..

[44] Connolly L.J., Bailey S.J., Krustrup P., Fulford J., Smietanka C., Jones A.M. (2017). Effects of self-paced interval and continuous training on health markers in women. Eur. J. Appl. Physiol..

[45] Cononie C.C., Graves J.E., Pollock M.L., Phillips M.I., Sumners C., Hagberg J.M. (1991). Effect of exercise training on blood pressure in 70- to 79-yr-old men and women. Med. Sci. Sports Exerc..

[46] Davies B., Daggett A. (1977). Responses of adult women to programmed exercise. Br. J. Sports Med..

[47] Delecluse C., Colman V., Roelants M., Verschueren S., Derave W., Ceux T., Eijnde B.O., Seghers J., Pardaens K., Brumagne S. (2004). Exercise programs for older men: Mode and intensity to induce the highest possible health-related benefits. Prev. Med..

[48] Dowdy D.B., Cureton K.J., Duval H.P., Ouzts H.G. (1985). Effects of Aerobic Dance on Physical Work Capacity, Cardiovascular Function and Body Composition of Middle-Aged Women. Res. Q. Exerc. Sport.

[49] Duey W.J., O’Brien W.L., Crutchfield A.B., Brown L.A., Williford H.N., Sharff-Olson M. (1998). Effects of exercise training on aerobic fitness in African-American females. Ethn. Dis..

[50] Duncan J.J., Farr J.E., Upton S.J., Hagan R.D., Oglesby M.E., Blair S.N. (1985). The effects of aerobic exercise on plasma catecholamines and blood pressure in patients with mild essential hypertension. JAMA.

[51] Ewart C.K., Young D.R., Hagberg J.M. (1998). Effects of school-based aerobic exercise on blood pressure in adolescent girls at risk for hypertension. Am. J. Public Health.

[52] Finucane F.M., Sharp S.J., Purslow L.R., Horton K., Horton J., Savage D.B., Brage S., Besson H., De Lucia Rolfe E., Sleigh A. (2010). The effects of aerobic exercise on metabolic risk, insulin sensitivity and intrahepatic lipid in healthy older people from the Hertfordshire Cohort Study: A randomised controlled trial. Diabetologia.

[53] Geenen D.L., Gilliam T.B., Crowley D., Moorehead-Steffens C., Rosenthal A. (1982). Echocardiographic measures in 6 to 7 year old children after an 8 month exercise program. Am. J. Cardiol..

[54] Goldberg M.J., Boutcher S.H., Boutcher Y.N. (2012). The effect of 4 weeks of aerobic exercise on vascular and baroreflex function of young men with a family history of hypertension. J. Hum. Hypertens..

[55] Gormley S.E., Swain D.P., High R., Spina R.J., Dowling E.A., Kotipalli U.S., Gandrakota R. (2008). Effect of intensity of aerobic training on VO_2_max. Med. Sci. Sports Exerc..

[56] Gossard D., Haskell W.L., Taylor C.B., Mueller J.K., Rogers F., Chandler M., Ahn D.K., Miller N.H., DeBusk R.F. (1986). Effects of low- and high-intensity home-based exercise training on functional capacity in healthy middle-aged men. Am. J. Cardiol..

[57] Gutin B., Owens S., Slavens G., Riggs S., Treiber F. (1997). Effect of physical training on heart-period variability in obese children. J. Pediatr..

[58] Hagberg J.M., Montain S.J., Martin W.H., Ehsani A.A. (1989). Effect of exercise training in 60- to 69-year-old persons with essential hypertension. Am. J. Cardiol..

[59] Halbert J.A., Silagy C.A., Finucane P.M., Withers R.T., Hamdorf P.A. (2000). Physical activity and cardiovascular risk factors: Effect of advice from an exercise specialist in Australian general practice. Med. J. Aust..

[60] Hamdorf P.A., Penhall R.K. (1999). Walking with its training effects on the fitness and activity patterns of 79–91 year old females. Aust. N. Z. J. Med..

[61] Hamdorf P.A., Withers R.T., Penhall R.K., Plummer J.L. (1993). A follow-up study on the effects of training on the fitness and habitual activity patterns of 60- to 70-year-old women. Arch. Phys. Med. Rehabil..

[62] O’Hartaigh B., Pahor M., Buford T.W., Dodson J.A., Forman D.E., Gill T.M., Group L.S. (2014). Physical activity and resting pulse rate in older adults: Findings from a randomized controlled trial. Am. Heart J..

[63] Hespel P., Lijnen P., Fagard R., Van Hoof R., Rosseneu M., Amery A. (1988). Changes in plasma lipids and apoproteins associated with physical training in middle-aged sedentary men. Am. Heart J..

[64] Hewitt J.A., Whyte G.P., Moreton M., van Someren K.A., Levine T.S. (2008). The effects of a graduated aerobic exercise programme on cardiovascular disease risk factors in the NHS workplace: A randomised controlled trial. J. Occup. Med. Toxicol..

[65] Heydari M., Freund J., Boutcher S.H. (2012). The effect of high-intensity intermittent exercise on body composition of overweight young males. J. Obes..

[66] Heydari M., Boutcher S.H. (2013). Rating of perceived exertion after 12 weeks of high-intensity, intermittent sprinting. Percept. Mot. Skills.

[67] Heydari M., Boutcher Y.N., Boutcher S.H. (2013). The effects of high-intensity intermittent exercise training on cardiovascular response to mental and physical challenge. Int. J. Psychophysiol..

[68] Higashi Y., Sasaki S., Sasaki N., Nakagawa K., Ueda T., Yoshimizu A., Kurisu S., Matsuura H., Kajiyama G., Oshima T. (1999). Daily aerobic exercise improves reactive hyperemia in patients with essential hypertension. Hypertension.

[69] Hill R.D., Storandt M., Malley M. (1993). The impact of long-term exercise training on psychological function in older adults. J. Gerontol..

[70] Hiruntrakul A., Nanagara R., Emasithi A., Borer K.T. (2010). Effect of endurance exercise on resting testosterone levels in sedentary subjects. Cent. Eur. J. Public Health.

[71] Hiruntrakul A., Nanagara R., Emasithi A., Borer K.T. (2010). Effect of once a week endurance exercise on fitness status in sedentary subjects. J. Med. Assoc. Thail..

[72] Hornstrup T., Wikman J.M., Fristrup B., Póvoas S., Helge E.W., Nielsen S.H., Helge J.W., Andersen J.L., Nybo L., Krustrup P. (2018). Fitness and health benefits of team handball training for young untrained women—A cross-disciplinary RCT on physiological adaptations and motivational aspects. J. Sport Health Sci..

[73] Jahromi S.R.A., Bahrani S.M.N., Hashemzadeh F., Safarpoor A. (2016). The effect of eight weeks rope-jump on leg muscles strength and resting heart rate of 10–11 years old female students. Res. J. Pharm. Biol. Chem. Sci..

[74] Jurca R., Church T.S., Morss G.M., Jordan A.N., Earnest C.P. (2004). Eight weeks of moderate-intensity exercise training increases heart rate variability in sedentary postmenopausal women. Am. Heart J..

[75] Karavirta L., Tulppo M.P., Laaksonen D.E., Nyman K., Laukkanen R.T., Kinnunen H., Hakkinen A., Hakkinen K. (2009). Heart rate dynamics after combined endurance and strength training in older men. Med. Sci. Sports Exerc..

[76] Karavirta L., Costa M.D., Goldberger A.L., Tulppo M.P., Laaksonen D.E., Nyman K., Keskitalo M., Hakkinen A., Hakkinen K. (2013). Heart rate dynamics after combined strength and endurance training in middle-aged women: Heterogeneity of responses. PLoS ONE.

[77] Kiens B., Jorgensen I., Lewis S., Jensen G., Lithell H., Vessby B., Hoe S., Schnohr P. (1980). Increased plasma HDL-cholesterol and apo A-1 in sedentary middle-aged men after physical conditioning. Eur. J. Clin. Investig..

[78] Kim E.S., Im J.A., Kim K.C., Park J.H., Suh S.H., Kang E.S., Kim S.H., Jekal Y., Lee C.W., Yoon Y.J. (2007). Improved insulin sensitivity and adiponectin level after exercise training in obese Korean youth. Obesity.

[79] Knoepfli-Lenzin C., Sennhauser C., Toigo M., Boutellier U., Bangsbo J., Krustrup P., Junge A., Dvorak J. (2010). Effects of a 12-week intervention period with football and running for habitually active men with mild hypertension. Scand. J. Med. Sci. Sports.

[80] Kokkinos P.F., Narayan P., Colleran J.A., Pittaras A., Notargiacomo A., Reda D., Papademetriou V. (1995). Effects of regular exercise on blood pressure and left ventricular hypertrophy in African-American men with severe hypertension. N. Engl. J. Med..

[81] Korshoj M., Lidegaard M., Skotte J.H., Krustrup P., Krause N., Sogaard K., Holtermann A. (2015). Does aerobic exercise improve or impair cardiorespiratory fitness and health among cleaners? A cluster randomized controlled trial. Scand. J. Work Environ. Health.

[82] Korshoj M., Lidegaard M., Krustrup P., Jorgensen M.B., Sogaard K., Holtermann A. (2016). Long Term Effects on Risk Factors for Cardiovascular Disease after 12-Months of Aerobic Exercise Intervention—A Worksite RCT among Cleaners. PLoS ONE.

[83] Krustrup P., Nielsen J.J., Krustrup B.R., Christensen J.F., Pedersen H., Randers M.B., Aagaard P., Petersen A.M., Nybo L., Bangsbo J. (2009). Recreational soccer is an effective health-promoting activity for untrained men. Br. J. Sports Med..

[84] Krustrup P., Hansen P.R., Randers M.B., Nybo L., Martone D., Andersen L.J., Bune L.T., Junge A., Bangsbo J. (2010). Beneficial effects of recreational football on the cardiovascular risk profile in untrained premenopausal women. Scand. J. Med. Sci. Sports.

[85] Krustrup P., Hansen P.R., Nielsen C.M., Larsen M.N., Randers M.B., Manniche V., Hansen L., Dvorak J., Bangsbo J. (2014). Structural and functional cardiac adaptations to a 10-week school-based football intervention for 9–10-year-old children. Scand. J. Med. Sci. Sports.

[86] Lamina S. (2010). Effects of continuous and interval training programs in the management of hypertension: A randomized controlled trial. J. Clin. Hypertens..

[87] Linder C.W., DuRant R.H., Mahoney O.M. (1983). The effect of physical conditioning on serum lipids and lipoproteins in white male adolescents. Med. Sci. Sports Exerc..

[88] Loimaala A., Huikuri H., Oja P., Pasanen M., Vuori I. (2000). Controlled 5-mo aerobic training improves heart rate but not heart rate variability or baroreflex sensitivity. J. Appl. Physiol..

[89] Mahdiabadi J. (2017). Central hemodynamic response to interval aerobic jogging in healthy male students. Pedag. Psychol. Med.-Biol. Probl. Phys. Train. Sports.

[90] Melanson E.L., Freedson P.S. (2001). The effect of endurance training on resting heart rate variability in sedentary adult males. Eur. J. Appl. Physiol..

[91] Menezes-Cabral R.L., Silva-Dantas P.M., Montenegro-Neto A.N., Knackfuss M.I. (2009). Effects of different types of training and life styles on anthropometric and cardiocirculatory markers in aging. Revista de Salud Publica.

[92] Meucci M., Cook C., Curry C.D., Guidetti L., Baldari C., Collier S.R. (2013). Effects of supervised exercise program on metabolic function in overweight adolescents. World J. Pediatr..

[93] Miyai N., Arita M., Miyashita K., Morioka I., Shiraishi T., Nishio I., Takeda S. (2002). Antihypertensive effects of aerobic exercise in middle-aged normotensive men with exaggerated blood pressure response to exercise. Hypertens. Res..

[94] Mogharnasi M., Eslami R., Behnam B. (2014). Effects of Endurance and Circuit Resistance Trainings on Lipid Profile, Heart Rate, and Hematological Parameters in Obese Male Students. Ann. Appl. Sport Sci..

[95] Mohr M., Nordsborg N.B., Lindenskov A., Steinholm H., Nielsen H.P., Mortensen J., Weihe P., Krustrup P. (2014). High-intensity intermittent swimming improves cardiovascular health status for women with mild hypertension. Biomed. Res. Int..

[96] Molmen-Hansen H.E., Stolen T., Tjonna A.E., Aamot I.L., Ekeberg I.S., Tyldum G.A., Wisloff U., Ingul C.B., Stoylen A. (2012). Aerobic interval training reduces blood pressure and improves myocardial function in hypertensive patients. Eur. J. Prev. Cardiol..

[97] Moreau K.L., Degarmo R., Langley J., McMahon C., Howley E.T., Bassett D.R., Thompson D.L. (2001). Increasing daily walking lowers blood pressure in postmenopausal women. Med. Sci. Sports Exerc..

[98] Morgan A.L., Tobar D.A., Snyder L. (2010). Walking toward a new me: The impact of prescribed walking 10,000 steps/day on physical and psychological well-being. J. Phys. Act. Health.

[99] Nemoto K., Gen-no H., Masuki S., Okazaki K., Nose H. (2007). Effects of high-intensity interval walking training on physical fitness and blood pressure in middle-aged and older people. Mayo Clin. Proc..

[100] Norris R., Carroll D., Cochrane R. (1992). The effects of physical activity and exercise training on psychological stress and well-being in an adolescent population. J. Psychosom. Res..

[101] Nualnim N., Parkhurst K., Dhindsa M., Tarumi T., Vavrek J., Tanaka H. (2012). Effects of swimming training on blood pressure and vascular function in adults >50 years of age. Am. J. Cardiol..

[102] Nybo L., Sundstrup E., Jakobsen M.D., Mohr M., Hornstrup T., Simonsen L., Bulow J., Randers M.B., Nielsen J.J., Aagaard P. (2010). High-intensity training versus traditional exercise interventions for promoting health. Med. Sci. Sports Exerc..

[103] Ortega J.F., Fernandez-Elias V.E., Hamouti N., Mora-Rodriguez R. (2013). Increased blood cholesterol after a high saturated fat diet is prevented by aerobic exercise training. Appl. Physiol. Nutr. Metab..

[104] Palmer L.K. (1995). Effects of a walking program on attributional style, depression, and self-esteem in women. Percept. Mot. Skills.

[105] Patterson S., Pattison J., Legg H., Gibson A.-M., Brown N. (2017). The impact of badminton on health markers in untrained females. J. Sports Sci..

[106] Pollock M.L., Miller H.S., Janeway R., Linnerud A.C., Robertson B., Valentino R. (1971). Effects of walking on body composition and cardiovascular function of middle-aged man. J. Appl. Physiol..

[107] Pollock M.L., Dimmick J., Miller H.S., Kendrick Z., Linnerud A.C. (1975). Effects of mode of training on cardiovascular function and body composition of adult men. Med. Sci. Sports.

[108] Pollock M.L., Dawson G.A., Miller H.S., Ward A., Cooper D., Headley W., Linnerud A.C., Nomeir M.M. (1976). Physiologic responses of men 49 to 65 years of age to endurance training. J. Am. Geriatr. Soc..

[109] Racil G., Coquart J.B., Elmontassar W., Haddad M., Goebel R., Chaouachi A., Amri M., Chamari K. (2016). Greater effects of high- compared with moderate-intensity interval training on cardio-metabolic variables, blood leptin concentration and ratings of perceived exertion in obese adolescent females. Biol. Sport.

[110] Ray C.A., Carter J.R. (2010). Effects of aerobic exercise training on sympathetic and renal responses to mental stress in humans. Am. J. Physiol. Heart Circ. Physiol..

[111] Richter C.M., Panigas T.F., Bundchen D.C., Dipp T., Belli K.C., Viecili P.R. (2010). Blood pressure reduction in hyper-reactive individuals after aerobic exercise. Arq. Brasil. Cardiol..

[112] Ruoti R.G., Troup J.T., Berger R.A. (1994). The effects of nonswimming water exercises on older adults. J. Orthop. Sports Phys. Ther..

[113] Sakai T., Ideishi M., Miura S., Maeda H., Tashiro E., Koga M., Kinoshita A., Sasaguri M., Tanaka H., Shindo M. (1998). Mild exercise activates renal dopamine system in mild hypertensives. J. Hum. Hypertens..

[114] Sakuragi S., Sugiyama Y. (2006). Effects of daily walking on subjective symptoms, mood and autonomic nervous function. J. Physiol. Anthropol..

[115] Schmidt J.F., Hansen P.R., Andersen T.R., Andersen L.J., Hornstrup T., Krustrup P., Bangsbo J. (2014). Cardiovascular adaptations to 4 and 12 months of football or strength training in 65- to 75-year-old untrained men. Scand. J. Med. Sci. Sports.

[116] Seals D.R., Hurley B.F., Hagberg J.M., Schultz J., Linder B.J., Natter L., Ehsani A.A. (1985). Effects of training on systolic time intervals at rest and during isometric exercise in men and women 61 to 64 years old. Am. J. Cardiol..

[117] Seals D.R., Reiling M.J. (1991). Effect of regular exercise on 24-hour arterial pressure in older hypertensive humans. Hypertension.

[118] Serwe K.M., Swartz A.M., Hart T.L., Strath S.J. (2011). Effectiveness of long and short bout walking on increasing physical activity in women. J. Womens Health.

[119] Shiotani H., Umegaki Y., Tanaka M., Kimura M., Ando H. (2009). Effects of aerobic exercise on the circadian rhythm of heart rate and blood pressure. Chronobiol. Int..

[120] Sijie T., Hainai Y., Fengying Y., Jianxiong W. (2012). High intensity interval exercise training in overweight young women. J. Sports Med. Phys. Fit..

[121] Spalding T.W., Lyon L.A., Steel D.H., Hatfield B.D. (2004). Aerobic exercise training and cardiovascular reactivity to psychological stress in sedentary young normotensive men and women. Psychophysiology.

[122] Stefanick M.L., Mackey S., Sheehan M., Ellsworth N., Haskell W.L., Wood P.D. (1998). Effects of diet and exercise in men and postmenopausal women with low levels of HDL cholesterol and high levels of LDL cholesterol. N. Engl. J. Med..

[123] Sugawara J., Akazawa N., Miyaki A., Choi Y., Tanabe Y., Imai T., Maeda S. (2012). Effect of endurance exercise training and curcumin intake on central arterial hemodynamics in postmenopausal women: Pilot study. Am. J. Hypertens..

[124] Suter E., Marti B., Tschopp A., Wanner H.U., Wenk C., Gutzwiller F. (1990). Effects of self-monitored jogging on physical fitness, blood pressure and serum lipids: A controlled study in sedentary middle-aged men. Int. J. Sports Med..

[125] Tanabe Y., Urata H., Kiyonaga A., Ikeda M., Tanaka H., Shindo M., Arakawa K. (1989). Changes in serum concentrations of taurine and other amino acids in clinical antihypertensive exercise therapy. Clin. Exp. Hypertens. A.

[126] Tanaka H., Reiling M.J., Seals D.R. (1998). Regular walking increases peak limb vasodilatory capacity of older hypertensive humans: Implications for arterial structure. J. Hypertens..

[127] Tanaka H., Bassett D.R., Howley E.T., Thompson D.L., Ashraf M., Rawson F.L. (1997). Swimming training lowers the resting blood pressure in individuals with hypertension. J. Hypertens..

[128] Tanaka H., Dale G.A., Bassett D.R. (1999). Influence of regular swimming on profile of mood states in obese subjects with essential hypertension. Jpn. J. Phys. Fit. Sports Med..

[129] Tanaka H., Bassett D.R., Howley E.T. (1997). Effects of swim training on body weight, carbohydrate metabolism, lipid and lipoprotein profile. Clin. Physiol..

[130] Tsai J.C., Chang W.Y., Kao C.C., Lu M.S., Chen Y.J., Chan P. (2002). Beneficial effect on blood pressure and lipid profile by programmed exercise training in Taiwanese patients with mild hypertension. Clin. Exp. Hypertens..

[131] Tsai J.C., Liu J.C., Kao C.C., Tomlinson B., Kao P.F., Chen J.W., Chan P. (2002). Beneficial effects on blood pressure and lipid profile of programmed exercise training in subjects with white coat hypertension. Am. J. Hypertens..

[132] Tsai J.C., Yang H.Y., Wang W.H., Hsieh M.H., Chen P.T., Kao C.C., Kao P.F., Wang C.H., Chan P. (2004). The beneficial effect of regular endurance exercise training on blood pressure and quality of life in patients with hypertension. Clin. Exp. Hypertens..

[133] Tully M.A., Cupples M.E., Chan W.S., McGlade K., Young I.S. (2005). Brisk walking, fitness, and cardiovascular risk: A randomized controlled trial in primary care. Prev. Med..

[134] Turner M.J., Spina R.J., Kohrt W.M., Ehsani A.A. (2000). Effect of endurance exercise training on left ventricular size and remodeling in older adults with hypertension. J. Gerontol. A Biol. Sci. Med. Sci..

[135] Urata H., Tanabe Y., Kiyonaga A., Ikeda M., Tanaka H., Shindo M., Arakawa K. (1987). Antihypertensive and volume-depleting effects of mild exercise on essential hypertension. Hypertension.

[136] Vance D.D., Chen G.L., Stoutenberg M., Myerburg R.J., Jacobs K., Nathanson L., Perry A., Seo D., Goldschmidt-Clermont P.J., Rampersaud E. (2014). Cardiac performance, biomarkers and gene expression studies in previously sedentary men participating in half-marathon training. BMC Sports Sci. Med. Rehabil..

[137] Wanderley F.A., Moreira A., Sokhatska O., Palmares C., Moreira P., Sandercock G., Oliveira J., Carvalho J. (2013). Differential responses of adiposity, inflammation and autonomic function to aerobic versus resistance training in older adults. Exp. Gerontol..

[138] Whitehurst M., Menendez E. (1991). Endurance Training in Older Women—Lipid and Lipoprotein Responses. Phys. Sportsmed..

[139] Winter M.M., van der Bom T., de Vries L.C., Balducci A., Bouma B.J., Pieper P.G., van Dijk A.P., van der Plas M.N., Picchio F.M., Mulder B.J. (2012). Exercise training improves exercise capacity in adult patients with a systemic right ventricle: A randomized clinical trial. Eur. Heart J..

[140] Wolfe L.A., Laprade A., Burggraf G.W., Norman R. (1992). Cardiac Responses of Young Women to Conditioning for a 10 Kilometer Race. Int. J. Sports Med.

[141] Wood P.D., Haskell W.L., Blair S.N., Williams P.T., Krauss R.M., Lindgren F.T., Albers J.J., Ho P.H., Farquhar J.W. (1983). Increased exercise level and plasma lipoprotein concentrations: A one-year, randomized, controlled study in sedentary, middle-aged men. Metabolism.

[142] Wood R.H., Reyes R., Welsch M.A., Favaloro-Sabatier J., Sabatier M., Matthew Lee C., Johnson L.G., Hooper P.F. (2001). Concurrent cardiovascular and resistance training in healthy older adults. Med. Sci. Sports Exerc..

[143] Wynne T.P., Frey M.A.B., Laubach L.L., Glueck C.J. (1980). Effect of a controlled exercise program on serum lipoprotein levels in women on oral contraceptives. Metabolism.

[144] Yamamoto K., Miyachi M., Saitoh T., Yoshioka A., Onodera S. (2001). Effects of endurance training on resting and post-exercise cardiac autonomic control. Med. Sci. Sports Exerc..

[145] Yoshizawa M., Maeda S., Miyaki A., Misono M., Saito Y., Tanabe K., Kuno S., Ajisaka R. (2009). Effect of 12 weeks of moderate-intensity resistance training on arterial stiffness: A randomised controlled trial in women aged 32–59 years. Br. J. Sports Med..

[146] Byrne H.K., Wilmore J.H. (2001). The effects of a 20-week exercise training program on resting metabolic rate in previously sedentary, moderately obese women. Int. J. Sport Nutr. Exerc. Metab..

[147] Cortez-Cooper M.Y., Anton M.M., Devan A.E., Neidre D.B., Cook J.N., Tanaka H. (2008). The effects of strength training on central arterial compliance in middle-aged and older adults. Eur. J. Cardiovasc. Prev. Rehabil..

[148] Deley G., Kervio G., Van Hoecke J., Verges B., Grassi B., Casillas J.M. (2007). Effects of a one-year exercise training program in adults over 70 years old: A study with a control group. Aging Clin. Exp. Res..

[149] Figueroa A., Park S.Y., Seo D.Y., Sanchez-Gonzalez M.A., Baek Y.H. (2011). Combined resistance and endurance exercise training improves arterial stiffness, blood pressure, and muscle strength in postmenopausal women. Menopause.

[150] Frye B., Scheinthal S., Kemarskaya T., Pruchno R. (2007). Tai Chi and Low Impact Exercise: Effects on the Physical Functioning and Psychological Well-Being of Older People. J. Appl. Gerontol..

[151] Masroor S., Bhati P., Verma S., Khan M., Hussain M.E. (2018). Heart Rate Variability following Combined Aerobic and Resistance Training in Sedentary Hypertensive Women: A Randomised Control Trial. Heart J..

[152] Ohkubo T., Hozawa A., Nagatomi R., Fujita K., Sauvaget C., Watanabe Y., Anzai Y., Tamagawa A., Tsuji I., Imai Y. (2001). Effects of exercise training on home blood pressure values in older adults: A randomized controlled trial. J. Hypertens..

[153] Stewart K.J., Bacher A.C., Turner K.L., Fleg J.L., Hees P.S., Shapiro E.P., Tayback M., Ouyang P. (2005). Effect of exercise on blood pressure in older persons: A randomized controlled trial. Arch. Intern. Med..

[154] Stewart K.J., Ouyang P., Bacher A.C., Lima S., Shapiro E.P. (2006). Exercise effects on cardiac size and left ventricular diastolic function: Relationships to changes in fitness, fatness, blood pressure and insulin resistance. Heart.

[155] Svendsen O.L., Hassager C., Christiansen C. (1993). Effect of an energy-restrictive diet, with or without exercise, on lean tissue mass, resting metabolic rate, cardiovascular risk factors, and bone in overweight postmenopausal women. Am. J. Med..

[156] Tsuda K., Yoshikawa A., Kimura K., Nishio I. (2003). Effects of mild aerobic physical exercise on membrane fluidity of erythrocytes in essential hypertension. Clin. Exp. Pharmacol. Physiol..

[157] Badrov M.B., Bartol C.L., DiBartolomeo M.A., Millar P.J., McNevin N.H., McGowan C.L. (2013). Effects of isometric handgrip training dose on resting blood pressure and resistance vessel endothelial function in normotensive women. Eur. J. Appl. Physiol..

[158] Baross A.W., Wiles J.D., Swaine I.L. (2013). Double-leg isometric exercise training in older men. Open Access J. Sports Med..

[159] Carter J.R., Ray C.A., Downs E.M., Cooke W.H. (2003). Strength training reduces arterial blood pressure but not sympathetic neural activity in young normotensive subjects. J. Appl. Physiol..

[160] Fripp R.R., Hodgson J.L. (1987). Effect of resistive training on plasma lipid and lipoprotein levels in male adolescents. J. Pediatr..

[161] Gelecek N., Ilcin N., Subasi S.S., Acar S., Demir N., Ormen M. (2012). The effects of resistance training on cardiovascular disease risk factors in postmenopausal women: A randomized-controlled trial. Health Care Women Int..

[162] Gerage A.M., Forjaz C.L., Nascimento M.A., Januario R.S., Polito M.D., Cyrino E.S. (2013). Cardiovascular adaptations to resistance training in elderly postmenopausal women. Int. J. Sports Med..

[163] Giannaki C.D., Aphamis G., Tsouloupas C.N., Ioannou Y., Hadjicharalambous M. (2016). An eight week school-based intervention with circuit training improves physical fitness and reduces body fat in male adolescents. J. Sports Med. Phys. Fit..

[164] Gurjão A.L.D., Gonçalves R., Carneiro N.H., Ceccato M., Jambassi Filho J.C., Gobbi S. (2013). Efeito do treinamento com pesos na pressão arterial de repouso em idosas normotensas. Rev. Bras. Med. Esporte.

[165] Harris K.A., Holly R.G. (1987). Physiological response to circuit weight training in borderline hypertensive subjects. Med. Sci. Sports Exerc..

[166] Ibrahim N.S., Muhamad A.S., Ooi F.K., Meor-Osman J., Chen C.K. (2018). The effects of combined probiotic ingestion and circuit training on muscular strength and power and cytokine responses in young males. Appl. Physiol. Nutr. Metab..

[167] Kanegusuku H., Queiroz A.C., Chehuen M.R., Costa L.A., Wallerstein L.F., Mello M.T., Ugrinowitsch C., Forjaz C.L. (2011). Strength and power training did not modify cardiovascular responses to aerobic exercise in elderly subjects. Braz. J. Med. Biol. Res..

[168] Lovell D.I., Cuneo R., Gass G.C. (2009). Resistance training reduces the blood pressure response of older men during submaximum aerobic exercise. Blood Press. Monit..

[169] Marinda F., Magda G., Ina S., Brandon S., Abel T., Ter Goon D. (2013). Effects of a mat pilates program on cardiometabolic parameters in elderly women. Pak. J. Med. Sci..

[170] Millar P.J., Levy A.S., McGowan C.L., McCartney N., MacDonald M.J. (2013). Isometric handgrip training lowers blood pressure and increases heart rate complexity in medicated hypertensive patients. Scand. J. Med. Sci. Sports.

[171] Miyachi M., Kawano H., Sugawara J., Takahashi K., Hayashi K., Yamazaki K., Tabata I., Tanaka H. (2004). Unfavorable effects of resistance training on central arterial compliance: A randomized intervention study. Circulation.

[172] Okamoto T., Masuhara M., Ikuta K. (2006). Effects of eccentric and concentric resistance training on arterial stiffness. J. Hum. Hypertens..

[173] Okamoto T., Masuhara M., Ikuta K. (2011). Effect of low-intensity resistance training on arterial function. Eur. J. Appl. Physiol..

[174] Shaw B.S., Gouveia M., McIntyre S., Shaw I. (2016). Anthropometric and cardiovascular responses to hypertrophic resistance training in postmenopausal women. Menopause.

[175] Stiller-Moldovan C., Kenno K., McGowan C.L. (2012). Effects of isometric handgrip training on blood pressure (resting and 24 h ambulatory) and heart rate variability in medicated hypertensive patients. Blood Press. Monit..

[176] Taylor A.C., McCartney N., Kamath M.V., Wiley R.L. (2003). Isometric training lowers resting blood pressure and modulates autonomic control. Med. Sci. Sports Exerc..

[177] Terra D.F., Mota M.R., Rabelo H.T., Bezerra L.M., Lima R.M., Ribeiro A.G., Vinhal P.H., Dias R.M., Silva F.M. (2008). Reduction of arterial pressure and double product at rest after resistance exercise training in elderly hypertensive women. Arq. Brasil. Cardiol..

[178] Thomas G.N., Hong A.W., Tomlinson B., Lau E., Lam C.W., Sanderson J.E., Woo J. (2005). Effects of Tai Chi and resistance training on cardiovascular risk factors in elderly Chinese subjects: A 12-month longitudinal, randomized, controlled intervention study. Clin. Endocrinol..

[179] Vincent K.R., Vincent H.K., Braith R.W., Bhatnagar V., Lowenthal D.T. (2003). Strength training and hemodynamic responses to exercise. Am. J. Geriatr. Cardiol..

[180] Wilmore J.H., Parr R.B., Girandola R.N., Ward P., Vodak P.A., Barstow T.J., Pipes T.V., Romero G.T., Leslie P. (1978). Physiological alterations consequent to circuit weight training. Med. Sci. Sports.

[181] Edwards K.M., Wilson K.L., Sadja J., Ziegler M.G., Mills P.J. (2011). Effects on blood pressure and autonomic nervous system function of a 12-week exercise or exercise plus DASH-diet intervention in individuals with elevated blood pressure. Acta Physiol..

[182] Garcia-Ortiz L., Grandes G., Sanchez-Perez A., Montoya I., Iglesias-Valiente J.A., Recio-Rodriguez J.I., Castano-Sanchez Y., Gomez-Marcos M.A., group P. (2010). Effect on cardiovascular risk of an intervention by family physicians to promote physical exercise among sedentary individuals. Rev. Esp. Cardiol..

[183] Hansen H.S., Froberg K., Hyldebrandt N., Nielsen J.R. (1991). A controlled study of eight months of physical training and reduction of blood pressure in children: The Odense schoolchild study. BMJ.

[184] Nogueira R.C., Weeks B.K., Beck B.R. (2014). An in-school exercise intervention to enhance bone and reduce fat in girls: The CAPO Kids trial. Bone.

[185] Rautela A. (2011). The effects of rhythmic activity on selected physiological and hysical fitness profile of school going girl’s. J. Phys. Educ. Sport.

[186] Walther C., Mende M., Gaede L., Muller U., Machalica K., Schuler G. (2011). Effects of daily physical exercise at school on cardiovascular risk—Results of a 2-year cluster-randomized study. Deut. Med. Wochenschr..

[187] Wong P.C., Chia M.Y., Tsou I.Y., Wansaicheong G.K., Tan B., Wang J.C., Tan J., Kim C.G., Boh G., Lim D. (2008). Effects of a 12-week exercise training programme on aerobic fitness, body composition, blood lipids and C-reactive protein in adolescents with obesity. Ann. Acad. Med. Singap..

[188] Lee M.S., Lee M.S., Choi E.S., Chung H.T. (2003). Effects of Qigong on blood pressure, blood pressure determinants and ventilatory function in middle-aged patients with essential hypertension. Am. J. Chin. Med..

[189] Li M., Fang Q., Li J., Zheng X., Tao J., Yan X., Lin Q., Lan X., Chen B., Zheng G. (2015). The Effect of Chinese Traditional Exercise-Baduanjin on Physical and Psychological Well-Being of College Students: A Randomized Controlled Trial. PLoS ONE.

[190] Sousa C.M., Goncalves M., Machado J., Efferth T., Greten T., Froeschen P., Greten H.J. (2012). Effects of qigong on performance-related anxiety and physiological stress functions in transverse flute music schoolchildren: A feasibility study. Zhong Xi Yi Jie He Xue Bao.

[191] Logghe I.H., Zeeuwe P.E., Verhagen A.P., Wijnen-Sponselee R.M., Willemsen S.P., Bierma-Zeinstra S.M., van Rossum E., Faber M.J., Koes B.W. (2009). Lack of effect of Tai Chi Chuan in preventing falls in elderly people living at home: A randomized clinical trial. J. Am. Geriatr. Soc..

[192] Nguyen M.H., Kruse A. (2012). The effects of Tai Chi training on physical fitness, perceived health, and blood pressure in elderly Vietnamese. Open Access J. Sports Med..

[193] Tsai J.C., Wang W.H., Chan P., Lin L.J., Wang C.H., Tomlinson B., Hsieh M.H., Yang H.Y., Liu J.C. (2003). The beneficial effects of Tai Chi Chuan on blood pressure and lipid profile and anxiety status in a randomized controlled trial. J. Altern. Complement. Med..

[194] Bezerra L.A., de Melo H.F., Garay A.P., Reis V.M., Aidar F.J., Bodas A.R., Garrido N.D., de Oliveira R.J. (2014). Do 12-week yoga program influence respiratory function of elderly women?. J. Hum. Kinet..

[195] Cheema B.S., Marshall P.W., Chang D., Colagiuri B., Machliss B. (2011). Effect of an office worksite-based yoga program on heart rate variability: A randomized controlled trial. BMC Public Health.

[196] Cohen D.L., Bloedon L.T., Rothman R.L., Farrar J.T., Galantino M.L., Volger S., Mayor C., Szapary P.O., Townsend R.R. (2011). Iyengar Yoga versus Enhanced Usual Care on Blood Pressure in Patients with Prehypertension to Stage I Hypertension: A Randomized Controlled Trial. Evid. Based Complement. Altern. Med..

[197] Hewett Z.L., Pumpa K.L., Smith C.A., Fahey P.P., Cheema B.S. (2018). Effect of a 16-week Bikram yoga program on perceived stress, self-efficacy and health-related quality of life in stressed and sedentary adults: A randomised controlled trial. J. Sci. Med. Sport.

[198] Kanojia S., Sharma V.K., Gandhi A., Kapoor R., Kukreja A., Subramanian S.K. (2013). Effect of yoga on autonomic functions and psychological status during both phases of menstrual cycle in young healthy females. J. Clin. Diagn. Res..

[199] Kim S., Bemben M.G., Bemben D.A. (2012). Effects of an 8-month yoga intervention on arterial compliance and muscle strength in premenopausal women. J. Sports Sci. Med..

[200] Krishna B.H., Pal P., Pal G.K., Balachander J., E J., Yerram S., Sridhar M.G., Gaur G.S. (2014). Effect of yoga therapy on heart rate, blood pressure and cardiac autonomic function in heart failure. J. Clin. Diagn. Res..

[201] Lau C., Yu R., Woo J. (2015). Effects of a 12-Week Hatha Yoga Intervention on Cardiorespiratory Endurance, Muscular Strength and Endurance, and Flexibility in Hong Kong Chinese Adults: A Controlled Clinical Trial. Evid. Based Complement. Altern. Med..

[202] Madanmohan, Mahadevan S.K., Balakrishnan S., Gopalakrishnan M., Prakash E.S. (2008). Effect of six weeks yoga training on weight loss following step test, respiratory pressures, handgrip strength and handgrip endurance in young healthy subjects. Indian J. Physiol. Pharmacol..

[203] McCaffrey R., Ruknui P., Hatthakit U., Kasetsomboon P. (2005). The effects of yoga on hypertensive persons in Thailand. Holist. Nurs. Pract..

[204] Mehrotra R., Phadke A., Kharche J., Pranita A., Joshi A. (2012). Effect of yoga on anxiety score and resting heart rate in young healthy individuals. Natl. J. Integr. Res. Med..

[205] Murugesan R., Govindarajulu N., Bera T.K. (2000). Effect of selected yogic practices on the management of hypertension. Indian J. Physiol. Pharmacol..

[206] Ray U.S., Mukhopadhyaya S., Purkayastha S.S., Asnani V., Tomer O.S., Prashad R., Thakur L., Selvamurthy W. (2001). Effect of yogic exercises on physical and mental health of young fellowship course trainees. Indian J. Physiol. Pharmacol..

[207] Sieverdes J.C., Mueller M., Gregoski M.J., Brunner-Jackson B., McQuade L., Matthews C., Treiber F.A. (2014). Effects of Hatha yoga on blood pressure, salivary alpha-amylase, and cortisol function among normotensive and prehypertensive youth. J. Altern. Complement. Med..

[208] Tew G.A., Howsam J., Hardy M., Bissell L. (2017). Adapted yoga to improve physical function and health-related quality of life in physically-inactive older adults: A randomised controlled pilot trial. BMC Geriatr..

[209] Thiyagarajan R., Pal P., Pal G.K., Subramanian S.K., Trakroo M., Bobby Z., Das A.K. (2015). Additional benefit of yoga to standard lifestyle modification on blood pressure in prehypertensive subjects: A randomized controlled study. Hypertens. Res..

[210] Udupa K., Madanmohan, Bhavanani A.B., Vijayalakshmi P., Krishnamurthy N. (2003). Effect of pranayam training on cardiac function in normal young volunteers. Indian J. Physiol. Pharmacol..

[211] Bahrainy S., Levy W.C., Busey J.M., Caldwell J.H., Stratton J.R. (2016). Exercise training bradycardia is largely explained by reduced intrinsic heart rate. Int. J. Cardiol..

[212] Tyagi A., Cohen M. (2016). Yoga and heart rate variability: A comprehensive review of the literature. Int. J. Yoga.

[213] Tadic M., Cuspidi C., Grassi G. (2018). Heart rate as a predictor of cardiovascular risk. Eur. J. Clin. Investig..

[214] Custodis F., Reil J.C., Schirmer S.H., Adam O., Mohlenkamp S., Laufs U., Bohm M. (2014). Heart rate: Clinical variable and risk marker. Dtsch. Med. Wochenschr..

